# *In silico* drug discovery pipelines targeting antibiotic resistance: from genomes to leads

**DOI:** 10.3389/fbinf.2026.1839606

**Published:** 2026-07-24

**Authors:** Rohan Gupta, Pallavi Singh, Karthikeyan Ravi, Mosleh Mohammad Abomughaid, Sorabh Lakhanpal, Ravi Sharma, Naveen Kumar, Niraj Kumar Jha

**Affiliations:** 1 Department of Biotechnology and Bioengineering, School of Biosciences and Technology, Galgotias University, Greater Noida, Uttar Pradesh, India; 2 Department of Biotechnology, Graphic Era Deemed to Be University, Dehradun Uttarakhand, India; 3 Centre for Herbal Pharmacology and Environmental Sustainability, Chettinad Hospital and Research Institute, Chettinad Academy of Research and Education, Kelambakkam, Tamil Nadu, India; 4 Department of Medical Laboratory Sciences, College of Applied Medical Sciences, University of Bisha, Bisha, Saudi Arabia; 5 School of Pharmaceutical Sciences, Lovely Professional University, Phagwara, Punjab, India; 6 School of Pharmacy, Sharda University, Greater Noida, Uttar Pradesh, India; 7 Department of Physics, School of Basic and Applied Sciences, Galgotias University, Greater Noida, Uttar Pradesh, India

**Keywords:** antibiotic resistance, genomic target identification, *in silico* drug discovery, machine learning, systems biology, systems pharmacology, virtual screening

## Abstract

Antimicrobial resistance (AMR) is currently one of the leading global health threats. The evolution of drug-resistant bacterial pathogens is rapid, and there is a growing number of bacterial pathogens that have developed resistance to multiple antibiotics, and the rate at which new antibiotics are being developed is lagging far behind these two issues. In addition, historical drug discovery processes rely on conducting traditional *in vitro*-based studies to determine new antibiotics to use in practice. However, this process is becoming increasingly constricted due to high costs of conducting traditional *in vitro* research, lengthy timeframes to bring products to market, a high attrition rate of research projects in traditional wet laboratory environments, and the need for more effective and efficient ways of developing new drugs. For this reason, Drug discovery continues to evolve from a wet lab-based approach to an *in silico* (i.e., computational) based approach, which takes advantage of the advances made in various fields, such as bacterial genomics, structural bioinformatics, machine learning (ML), and systems biology to enable researchers to rationally design, discover, and develop new antibiotics to combat drug-resistant pathogens. This review aims to provide a comprehensive and critical overview of contemporary *in silico* antibiotic discovery strategies and their potential to accelerate the development of novel, resistance-resilient, and clinically relevant antimicrobial agents. It examines genome-informed approaches ranging from genomic data generation, resistome analysis, and computational target identification to structure-based drug design, ligand-based and fragment-based discovery, drug repurposing, and the expanding applications of ML and artificial intelligence (AI) in activity prediction, *de novo* antibiotic design, and resistance evolution modeling. The review also highlights the importance of *in silico* ADMET prediction in lead optimization and discusses representative case studies demonstrating successful translation of computational predictions into experimental validation. Overall, the integration of digital-first, data-driven, and genome-guided discovery pipelines with experimental validation offers a powerful framework to overcome current challenges in antibiotic development and represents a promising strategy for addressing the global threat of AMR. Lastly, the review was conducted using a structured literature search across major biomedical and computational databases with emphasis on experimentally validated case studies and translational relevance.

## Graphical Abstract

**Figure:**
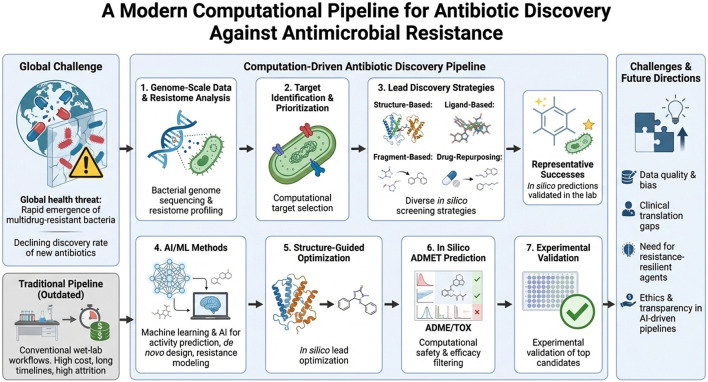
Overview of a modern computational pipeline for antibiotic discovery against antimicrobial resistance (AMR): The workflow begins with genome-scale bacterial sequencing and resistome analysis to identify potential drug targets. Computational prioritization of essential and druggable proteins guides lead discovery through structure-based, ligand-based, fragment-based, and drug-repurposing strategies. Artificial intelligence and machine learning support activity prediction, resistance modeling, and *de novo* compound design. Promising candidates undergo structure-guided optimization followed by *in silico* ADMET evaluation for safety and pharmacokinetic properties. Top compounds are then experimentally validated in laboratory assays. The framework highlights key challenges, including data bias, translation gaps, ethical considerations, and the need.

## Highlights

- Integrates AI-driven computational pipelines with experimental antibiotic discovery
- Combines structure-based, fragment-based, and repurposing strategies
- Applies machine learning for activity, ADMET, and resistance prediction
- Validates *in silico* hits through *in vitro* and *in vivo* models
- Addresses PK/PD discordance and translational bottlenecks in antibiotics
- Promotes iterative computational-experimental feedback for clinical translation

## 1 Introduction

**FIGURE 1:**
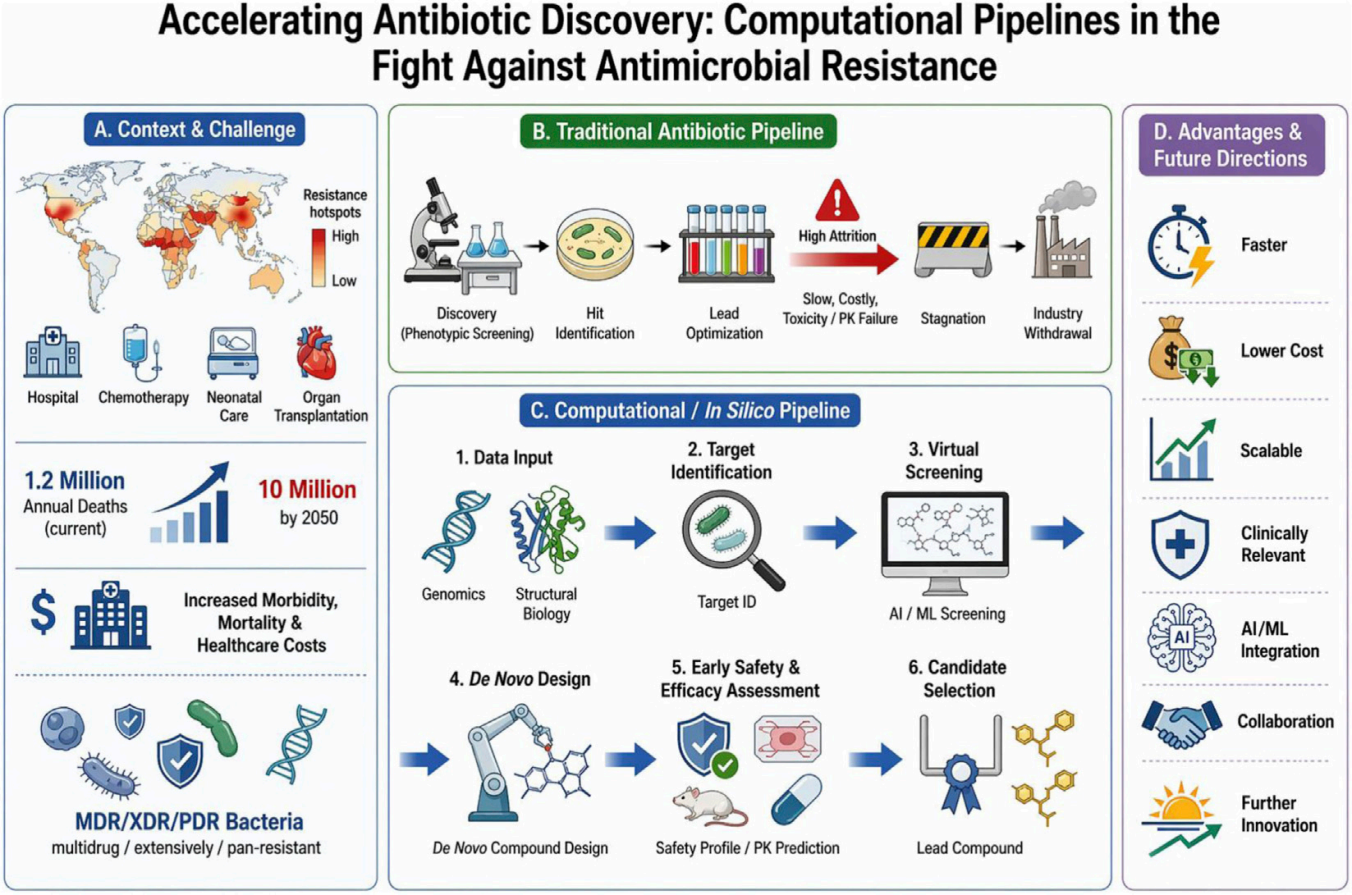
Overview of computational pipelines accelerating antibiotic discovery against antimicrobial resistance (AMR). Panel A shows the global burden of AMR, including rising morbidity, healthcare costs, and deaths from MDR, XDR, and PDR bacteria. Panel B highlights limitations of traditional discovery, including slow workflows and high attrition. Panel C illustrates the *in silico* pipeline from genomic data to candidate selection using AI/ML. Panel D summarizes advantages including speed, lower cost, scalability, clinical relevance, and innovation in antimicrobial development.

Antibiotic resistance has emerged as one of the most critical global health threats of the twenty-first century, undermining decades of progress in infectious disease control and modern medicine. The rapid spread of multidrug-resistant (MDR), extensively drug-resistant (XDR), and pan-resistant bacterial pathogens has rendered many first-line and last-resort antibiotics increasingly ineffective, leading to rising morbidity, mortality, and healthcare costs worldwide (Assefa et al., 2025). Antibiotic resistance now compromises not only the treatment of common bacterial infections but also the safety of complex medical interventions such as surgery, chemotherapy, organ transplantation, and neonatal care, all of which depend on effective antimicrobial prophylaxis (Teillant et al., 2015). The World Health Organization (WHO) has identified antimicrobial resistance (AMR) as a top ten global public health threat and has prioritized a list of high-risk bacterial pathogens, including *Acinetobacter baumannii*, *Pseudomonas aeruginosa*, *carbapenem-resistant Enterobacterales*, methicillin-resistant *Staphylococcus aureus* (MRSA), and drug-resistant *Mycobacterium tuberculosis* (https://www.who.int/publications/i/item/9789240093461) (May 2024) (Sati et al., 2025). The significance of antimicrobial drug development extends far beyond the treatment of individual infections; it underpins the entire foundation of modern healthcare systems. Effective antimicrobial agents are essential not only for controlling life-threatening bacterial diseases but also for enabling safe surgical procedures, cancer chemotherapy, intensive care management, transplantation, and maternal and neonatal medicine (Zavaleta-Monestel et al., 2025). Without a continuous pipeline of novel antibiotics, the effectiveness of these medical interventions is severely compromised, increasing the risk of postoperative infections, opportunistic infections, and healthcare-associated outbreaks. Furthermore, antimicrobial drug development plays a central role in global health security by preventing the spread of resistant pathogens across communities, hospitals, and international borders (Karnwal et al., 2025). The development of new antibiotics with novel mechanisms of action, improved pharmacokinetic profiles, reduced toxicity, and lower susceptibility to resistance evolution is therefore not merely a pharmaceutical objective but a strategic necessity for sustaining public health, economic stability, and future medical innovation (Machado and Sousa, 2025). Recent epidemiological analyses estimate that AMR directly causes more than 1.2 million deaths annually, with projections suggesting that this number could rise to 10 million deaths per year by 2050 if effective interventions are not developed. The burden is disproportionately high in low- and middle-income countries, where limited access to diagnostics, surveillance, and novel therapeutics accelerates the spread of resistant pathogens (Naghavi et al., 2024). Beyond mortality, the economic and healthcare impact of antibiotic resistance is profound. AMR is associated with prolonged hospital stays, increased use of expensive second- or third-line therapies, higher rates of treatment failure, and increased risk of complications. Moreover, global economic models predict cumulative losses of trillions of dollars over the coming decades due to reduced productivity, increased healthcare expenditure, and strain on healthcare systems (Naghavi et al., 2024). Despite this escalating crisis, the pace of novel antibiotic development has remained alarmingly slow, creating a widening gap between clinical need and therapeutic availability. A major contributor to this gap is the failure of traditional antibiotic discovery pipelines. Conventional wet-lab-based discovery strategies, which rely heavily on empirical high-throughput screening of chemical libraries against bacterial growth or enzymatic targets, have suffered from diminishing returns (Theuretzbacher et al., 2020; Anderson et al., 2023). Many pharmaceutical companies have withdrawn from antibiotic research due to high development costs, low commercial incentives, and frequent late-stage failures. Even when promising compounds are identified, high attrition rates during preclinical and clinical development, which are often due to toxicity, poor pharmacokinetics, or rapid emergence of resistance that significantly limit successful translation to approved drugs (Ahmed et al., 2024; Piddock et al., 2024a). Likewise, traditional wet-lab drug discovery is also inherently time- and resource-intensive. The process from initial screening to clinical approval typically spans more than a decade and requires substantial financial investment. Furthermore, many compounds that demonstrate potent *in vitro* antibacterial activity fail to translate into effective *in vivo* therapies due to poor target engagement, limited tissue penetration, or susceptibility to bacterial resistance mechanisms, such as efflux pumps and enzymatic degradation (DiMasi et al., 2016; Lewis, 2020). These limitations highlight the urgent need for more efficient, predictive, and rational approaches to antibiotic discovery. In this context, *in silico* drug discovery has emerged as a powerful and increasingly indispensable strategy in antimicrobial research. Recent advances in next-generation sequencing (NGS) have significantly improved the understanding of bacterial genomes and accelerated the application of *in silico* approaches in antibiotic discovery. Whole-genome sequencing now allows rapid and comprehensive characterization of bacterial genomes, including the identification of essential genes, virulence factors, resistance determinants, resistomes, and pan-genomes, providing deeper insights into pathogen evolution, strain diversity, and antimicrobial resistance mechanisms (Lewis, 2013; Thomas et al., 2018; Ingle et al., 2021). Genome-scale data have become a critical foundation for computational target discovery, enabling researchers to distinguish conserved pathogen-specific targets from host homologs and thereby improve target selectivity and reduce off-target toxicity. *In silico* approaches refer to computational strategies that use biological, chemical, and structural data to identify, prioritize, and optimize potential antimicrobial targets and lead compounds before laboratory validation (Bravo-Santano et al., 2019; Ta et al., 2026). These methods have become increasingly important because traditional empirical screening approaches are often expensive, time-consuming, and associated with high failure rates. The integration of genomic information with computational modeling allows a more rational and mechanism-driven strategy for antibiotic discovery (Wu et al., 2025). Parallel progress in structural biology, particularly cryo-electron microscopy and AI-based protein structure prediction tools such as AlphaFold, has greatly expanded the availability of high-resolution three-dimensional structures of bacterial proteins. This structural information is essential for understanding target function and for enabling structure-based drug design (Chakraborty et al., 2026). At the same time, advances in computational power, algorithm development, and ML have enhanced the ability to process complex biological datasets and explore vast chemical libraries with high speed and accuracy. These developments have shifted antibiotic discovery from conventional trial-and-error methods toward precision-guided therapeutic design (Jumper et al., 2021; Yakobi and Nwodo, 2025). Modern *in silico* pipelines now support systematic identification and prioritization of essential bacterial targets, prediction of druggability and selectivity, structure-based (SBVS) and ligand-based virtual screening (LBVS), fragment-based discovery, *de novo* compound design, and early evaluation of absorption, distribution, metabolism, excretion, and toxicity (ADMET) properties (Panickar et al., 2025). Importantly, these computational approaches can be iteratively integrated with experimental validation, significantly reducing development costs, shortening discovery timelines, and increasing the probability of identifying clinically viable antimicrobial lead compounds (Dai et al., 2021). Given the growing challenge of AMR and the limitations of conventional antibiotic discovery approaches, there is an urgent need for more efficient, precise, and scalable strategies for identifying new antimicrobial agents. In this context, *in silico* drug discovery has emerged as a powerful approach that integrates genomic data, computational biology, structural bioinformatics, and AI to accelerate the development of novel antibiotics (Stokes et al., 2020). This review focuses on the role of genome-informed computational pipelines in addressing antibiotic resistance by connecting bacterial genome analysis with rational lead compound discovery and optimization. It examines how genomic and systems-level analyses support target identification and prioritization, how structural modeling and bioinformatics facilitate mechanism-driven drug design, and how virtual screening, ML, and ADMET prediction improve lead selection and optimization. By critically evaluating recent methodological advances and representative validation studies, this review aims to provide a clearer understanding of both the opportunities and current limitations of computational antibiotic discovery and to outline future directions for developing robust, clinically relevant, and resistance-resilient antimicrobial pipelines (Figure 1).

### 1.1 Literature search strategy and methodological framework

This review was conducted using a structured narrative review approach to comprehensively summarize recent advances in *in silico* antibiotic discovery pipelines targeting AMR. A systematic literature search was performed across multiple electronic databases, including PubMed, Scopus, Web of Science, Google Scholar, ScienceDirect, and Embase, to identify relevant peer-reviewed articles published between January 2005 and May 2026. Additional landmark studies published before this period were included when considered foundational to the field, particularly for classical computational methods such as molecular docking, QSAR modeling, and genome-based target identification. The search strategy combined Medical Subject Headings (MeSH) terms and keyword-based Boolean operators using combinations of terms such as: “antimicrobial resistance,” “antibiotic resistance,” “*in silico* drug discovery,” “virtual screening,” “molecular docking,” “machine learning,” “artificial intelligence,” “genome-guided drug discovery,” “resistome analysis,” “pan-genomics,” “target identification,” “structure-based drug design,” “ligand-based drug design,” “fragment-based drug discovery,” “drug repurposing,” and “ADMET prediction.” Search strings were adapted according to the indexing requirements of individual databases. Studies were included if they met the following criteria: (i) original research articles, systematic reviews, and high-quality review articles published in peer-reviewed journals; (ii) studies focused on computational approaches for antibiotic discovery, antimicrobial resistance mechanisms, bacterial target identification, or translational validation of *in silico* predictions; (iii) studies involving experimentally validated case studies of computationally predicted antibacterial compounds; and (iv) articles published in English. Studies were excluded if they were conference abstracts, editorials, opinion articles, duplicate reports, studies lacking sufficient methodological detail, or reports focused exclusively on antifungal, antiviral, or antiparasitic drug discovery without relevance to antibacterial resistance. To improve scientific rigor, priority was given to studies with strong experimental validation, including biochemical assays, microbiological validation, animal infection models, and clinically relevant translational evidence. Particular emphasis was placed on landmark case studies, such as halicin discovery, β-lactamase inhibitor development, efflux pump inhibitor screening, and AI-assisted antibiotic design. Quality appraisal was performed based on methodological robustness, reproducibility of computational workflows, availability of validation experiments, and translational relevance to clinical antimicrobial development. The final manuscript synthesizes evidence from genomic studies, structural bioinformatics, molecular docking, machine learning pipelines, ADMET optimization, and translational case studies to provide an integrated perspective on the current status, limitations, and future directions of computational antibiotic discovery against AMR.

## 2 From whole-genome sequencing to pan-genomics: mapping the resistance landscape

**FIGURE 2:**
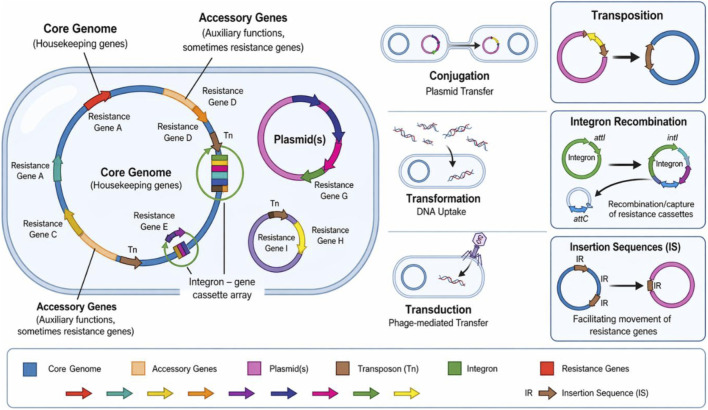
Schematic illustration of genetic organization and horizontal transfer mechanisms driving antibiotic resistance dissemination in bacteria. Resistance genes occur within chromosomal accessory regions or mobile genetic elements including plasmids, transposons, integrons, and insertion sequences. These elements promote gene capture, recombination, and mobilization. Horizontal gene transfer occurs via conjugation, transformation, and transduction, enabling rapid spread of multidrug resistance among bacterial populations and clinically important pathogenic strains.

The rising global threat of antibiotic-resistant pathogens at an increasingly rapid rate is, at its core, a genomic phenomenon driven by the evolving dynamics of bacterial genome evolution under selective pressure. Recent breakthroughs in high-throughput sequencing and comparative genomics have fundamentally shifted our paradigm of resistance from a set of isolated genes to a systems-level phenomenon of bacterial populations (McInerney et al., 2017; Hu et al., 2025). These genomic findings have become the central paradigm of modern *in silico* drug development pipelines, seeking to uncover new targets, resistance-circumventing strategies, and precision antimicrobials. This section will explore how the integration of bacterial genome sequencing, resistome analysis, mechanism-based annotation, and pan-genomic analysis collectively shapes the genomic landscape of antibiotic resistance (Figure 2).

### 2.1 Bacterial genome sequencing and resistomes profiling

Whole-genome sequencing (WGS) has come to be considered crucial for the high-resolution analysis of antibiotic resistance. Short-read sequencing platforms like Illumina are highly accurate and deep, making them ideal for the identification of single nucleotide polymorphisms (SNPs), small insertions/deletions, and known resistance genes in large-scale bacterial populations (Almaghrabi et al., 2024). However, assemblies generated from Illumina reads often break up repetitive segments, plasmids, and mobile genetic elements (MGEs), which are known to be rich in resistance genes. Additionally, long-read sequencing platforms like Oxford Nanopore and Pacific Biosciences (PacBio) sequencing overcome these challenges by producing reads that range from kilo-to megabases, allowing for the closure of the genome, the reconstruction of plasmids, and the precise mapping of resistance genes either within the chromosomal or extrachromosomal background (Berbers et al., 2020; Zhao et al., 2023; Maddamsetti et al., 2024; Bloemen et al., 2025). Two-platform assemblies that combine the high accuracy of Illumina sequencing with the long-range information of long-read sequencing are increasingly being viewed as the standard for resistome profiling studies, especially in MDR and XDR bacteria (Berbers et al., 2020; Sierra et al., 2024; Saticioglu et al., 2026). Moreover, resistome profiling relies on the use of carefully maintained databases that contain information on known antibiotic resistance genes (ARGs) and their mutations. Some of the most widely used databases include the Comprehensive Antibiotic Resistance Database (CARD) (https://card.mcmaster.ca/), which provides information on molecular mechanisms, resistance ontologies, and protein homology models; ResFinder (https://genepi.food.dtu.dk/resfinder), which focuses on clinically important acquired resistance genes; and ARG-ANNOT (https://www.mediterranee-infection.com/acces-ressources/base-de-donnees/arg-annot-2/), which emphasizes the annotation of resistance genes in a wide range of bacterial species (Gupta et al., 2014; Alcock et al., 2023; Manzoor et al., 2025). These databases enable the automated detection of resistance determinants from whole genome assemblies or sequencing reads, thus facilitating large-scale surveillance and comparative analysis. However, instead of just presence/absence information, comparative genomics of resistant and susceptible strains provides more information on the evolution of resistance. Further, genome-wide association studies (GWAS) and phylogenetically informed comparisons demonstrate that resistance is usually generated by multiple point mutations, gene duplications, regulatory mutations, and structural variations rather than a single genetic event. *In silico* drug discovery is facilitated by such comparisons, which allow for the distinction of conserved resistance-associated variations from strain-specific noise, thus facilitating the prioritization of targets that are both mechanistically relevant and conserved (Palmieri et al., 2024; Gontjes et al., 2025).

### 2.2 Mechanisms encoded in resistance genomes

Resistance genomes encode a wide range of molecular mechanisms that cumulatively contribute to the reduced efficacy of antibiotics. These mechanisms are often modular, combinatorial, and context-dependent, emphasizing the need for genome-informed therapeutic strategies (Wittenbach et al., 2026; Colac et al., 2026). For instance, enzymatic inactivation of drugs is one of the most well-studied resistance mechanisms. Genes that code for β-lactamases, aminoglycoside-modifying enzymes, chloramphenicol acetyltransferases, and macrolide esterases encode chemical modifications that make antibiotics ineffective. Genome studies have shown that these enzyme families are highly diversified, including extended-spectrum β-lactamases (ESBLs) and carbapenemases, making it difficult to design inhibitors (Pradier and Bedhomme, 2023; Hemmings et al., 2025; Sudeep and Han, 2025). From a computational perspective, enzyme-based resistance mechanisms are ideal targets for structure-based drug design, as detailed structural information and conserved catalytic regions facilitate the rational design of inhibitors. Target modification is another prominent genomic resistance mechanism, which is often achieved through point mutations or post-translational modification (PTMs) enzymes (Fyfe et al., 2016; Zárate et al., 2018). Further, modifications in ribosomal RNA, DNA gyrase, RNA polymerase, or penicillin-binding proteins decrease the affinity of antibiotics to their targets without affecting essential cellular processes. Resistance genomes often encode methyltransferases or harbor mutational hotspots that provide cross-resistance to multiple classes of drugs. These mechanisms highlight the need for the integration of genomic variation information into *in silico* docking and molecular dynamics simulations, as small structural variations can significantly affect drug-target interactions (Unissa et al., 2016; Zhou et al., 2022; Lagutkin et al., 2022; Kim S. et al., 2025). Additionally, efflux pumps and permeability barriers are ubiquitous mechanisms of multidrug resistance. Resistance genomes tend to harbor genes that encode resistance-nodulation-cell division, major facilitator superfamily, ATP-binding cassette (ABC), and small multidrug resistance (SMR) transporters. In Gram-negative bacteria, these efflux pumps act synergistically with decreased outer membrane permeability, due to either porin loss or modification, to limit intracellular drug concentrations (Nikaido and Pagès, 2012; Fernández and Hancock, 2012; Chiang and Dekker, 2024; Detweiler and Ilyas, 2025). Genomic studies suggest that efflux mechanisms are often controlled by complex transcriptional regulatory networks, suggesting that resistance can arise from mutations within regulatory regions as well as gene transfer events. The *in silico* discovery of efflux inhibitors or permeability-potentiating agents increasingly requires genome-informed modeling of membrane proteins and regulatory networks. Moreover, biofilm-associated resistance is a higher-order phenotype that is indirectly specified by resistance genomes (Blair et al., 2015; Trampari et al., 2023; Ren et al., 2024; Zack et al., 2024; Laborda et al., 2024). Recent evidence shows that bacterial persistence is strongly shaped by host-pathogen interactions. Macrophage-derived ROS can induce aggresome formation in intracellular *Salmonella*, creating dormant persisters with high antibiotic tolerance despite lacking classical resistance mutations. This highlights the need to include persistence biology and host-induced stress adaptation in computational antimicrobial target discovery (Chen X. et al., 2025). Additionally, genes involved in extracellular matrix synthesis, quorum sensing, stress responses, and metabolic dormancy act in concert to facilitate biofilm development, resulting in antibiotic tolerance in the absence of resistance mutations. Comparative genomics studies show that biofilm-associated genes are overrepresented in isolates from chronic infections, emphasizing the need to target biofilm-specific pathways. For *in silico* discovery, this necessitates a transition from gene-level targets to network- and systems-level modeling strategies (Liu H. et al., 2024; Blaznik et al., 2025).

### 2.3 Pan-genomics and strain-level heterogeneity

One of the most important findings in bacterial genomics is that antimicrobial resistance cannot be adequately explained by single reference genomes. Pan-genomic analysis, which divides the genome into core and accessory parts, has revolutionized the understanding of resistance evolution and drug targets. The core genome contains genes that code essential cellular processes and is usually conserved across a species (Wyres and Holt, 2016; Nesporova et al., 2025). Core gene targets have the potential for broad-spectrum efficacy and a lower probability of rapid resistance development. However, resistance often evolves through modifications or regulatory changes in core pathways. Thus, there is a need to carefully evaluate evolutionary constraints and compensatory mechanisms. The accessory genome, on the other hand, is very dynamic and contains a high concentration of ARGs, virulence factors, and adaptive functions (Maisnier-Patin and Andersson, 2004). The accessory genome is usually composed of genes that are present in mobile genetic elements, such as plasmids, transposons, integrons, and bacteriophages, which facilitate the rapid dissemination of resistance by horizontal gene transfer (HGT). Genomic studies have shown that the evolution of important resistance phenotypes usually occurs by HGT, which is faster than clonal evolution, thus challenging the traditional understanding of pathogen evolution (Koh et al., 2025; Sun et al., 2026). In terms of drug discovery, pan-genomics reveals the basic trade-off between broad-spectrum and narrow-spectrum drugs. Broad-spectrum drugs targeting the conserved core often face the risk of disrupting commensal microbiota and promoting resistance in various species. Narrow-spectrum or strain-specific drugs based on accessory genome attributes provide precise targeting but rely on reliable genomic diagnoses and may have a limited market reach (Rea et al., 2011; Demeke et al., 2021; Serral et al., 2021). Further, *in silico* drug discovery pipelines are increasingly leveraging pan-genomic information to discover pathogen-specific weaknesses and avoid targets prone to horizontal transfer. Further, strain-level variability further adds complexity to resistance prediction and target validation. Even for strains of the same species, there may be distinct resistance profiles, regulatory, and metabolic patterns. Pan-genomics combined with transcriptomics, proteomics, and phenotypic information can better model resistance patterns and facilitate the development of adaptive and context-aware therapeutic approaches (Fan et al., 2024; Chen Y. et al., 2025; Jiang et al., 2025; Ghosh et al., 2025).

Taken together, the combination of bacterial genome sequencing, mechanistic annotation, and pan-genomic analysis provides the foundational knowledge required to move from descriptive resistance surveillance to predictive, genome-guided drug discovery. Further, the transition from pan-genomic analysis to target prioritization represents a critical decision-making funnel in modern antimicrobial drug discovery. While whole-genome sequencing and resistome profiling identify the full spectrum of resistance determinants and adaptive genomic features, pan-genomics further distinguishes conserved core genes from highly variable accessory elements, enabling a more strategic selection of therapeutic targets (Sun et al., 2025). Core genome analysis helps identify evolutionarily conserved essential genes with broad-spectrum potential and lower susceptibility to rapid mutational escape, whereas accessory genome analysis reveals strain-specific vulnerabilities, virulence determinants, and horizontally transferred resistance elements that may support precision or narrow-spectrum therapies (Ibrahim et al., 2026). This genomic stratification must then be integrated with essentiality mapping, metabolic dependency analysis, protein interaction networks, and host-pathogen selectivity filters to prioritize targets that are not only biologically indispensable but also structurally druggable and clinically translatable (Zhang X. et al., 2024). Thus, target prioritization is not a single-step selection process, but a progressive refinement pipeline that moves from genomic landscape interpretation to mechanism-driven identification of high-value antimicrobial intervention points.

## 3 Target identification and prioritization using bioinformatics

Rational antibiotic discovery begins with the identification of microbial targets that are essential for pathogen survival or virulence and druggable, i.e., chemically tractable and selectively active against the pathogen without host toxicity. Modern bioinformatics facilitates this process at scale by integrating genome-wide experiments with systems biology, thus moving from single gene intuition to network-guided, mechanism-driven target selection (Xu et al., 2011; Serral et al., 2021; Warrier et al., 2022). Further, genetic approaches to foundational research, such as systematic knockout and defined deletion libraries (e.g., the *Escherichia coli* Keio collection), provided a comprehensive map of loss-of-function for essential genes. These maps were later improved by the integration of high-throughput transposon sequencing (Tn-seq) and deep sequencing to generate genome-wide fitness maps under a variety of host-relevant conditions. Pioneering Tn-seq studies in pathogens, such as *Salmonella* and *Streptococcus* revealed conditional essentiality in response to stress and antibiotics, highlighting the importance of context-dependent vulnerabilities not captured by static essential gene sets (Baba et al., 2006; Lourdault et al., 2016; Leshchiner et al., 2022). This idea was later generalized to important human pathogens, including *M. tuberculosis* and *Acinetobacter baumannii*. Comparison of Tn-seq data sets across strains and species allows the detection of conserved essential loci with low evolutionary tolerance, high-value targets that are less likely to develop resistance rapidly, as well as lineage-specific vulnerabilities amenable to narrow-spectrum approaches (Tateishi et al., 2020; Bai et al., 2021; Bosch et al., 2021). In addition to essentiality mapping, approaches based on pairwise genetic perturbations, chemical-genetic interaction mapping, and CRISPRi screens have shown that the simultaneous inactivation of gene pairs can be lethal even if individual genes are not essential, thus expanding the targetable space and justifying combination approaches to limit resistance emergence (Klobucar and Brown, 2018; Jiang et al., 2020; Liu X. et al., 2024). Moreover, systems biology refines these results using protein-protein interaction (PPI) maps, regulatory maps, and genome-scale metabolic models (GEMs), which reveal hubs and metabolic chokepoints whose inactivation triggers systemic failure; experimentally validated flux balance analysis (FBA) and fluxomics-integrated GEMs have successfully predicted lethal knockouts and chokepoint enzymes in pathogens, such as *P. aeruginosa* and *M. tuberculosis*, while joint PPI-GEM approaches have lowered false positives by selecting targets that affect both metabolic flux and essential multiprotein complexes (Oberhardt et al., 2008; Sertbas and Ulgen, 2020; Zhu Y. et al., 2022; Dahal et al., 2023). Network hub and chokepoint analyses have been validated by the bactericidal activity induced by inhibition of enzymes involved in particular folate or lipid biosynthesis pathways *in vitro* and *in vivo*. In addition to bactericidal approaches, modeling of host-pathogen interactions and the identification of virulence-related targets via dual RNA-Seq, proteomics, and affinity-binding interaction maps inform anti-virulence strategies that disable secretion systems, adhesins, or quorum-sensing systems, reducing selective pressure for resistance while maintaining *in vivo* efficacy (Westermann et al., 2017; Oyelade et al., 2019; Narad and Bansal, 2021; Bai et al., 2023). Downstream target selection is optimized by druggability analysis, which combines biological importance with structural and chemical tractability; the recent surge in high-resolution structural data from cryo-EM, X-ray crystallography, and AI-driven predictions (AlphaFold) has enabled near-complete structural profiling of the proteomes of many bacterial species, allowing docking and binding pocket prediction algorithms, such as FTMap (https://ftmap.bu.edu/serverhelp.php) or DoGSite (https://proteins.plus/2ozr#dogsite3) to inform ligandability, with fragment-based screening and biochemical validation confirming the enhanced hit rate of targets selected according to integrated essentiality and druggability criteria (Blundell et al., 2006; Volkamer et al., 2012; Erlanson et al., 2016; Tondi, 2021; Jumper et al., 2021). Finally, the issue of selectivity for human orthologs is resolved by comparative structural and orthology studies in combination with experimental cross-reactivity and toxicity assays, enabling early target de-prioritization for promiscuous targets and minimizing late-stage failures, collectively illustrating the role of integrated bioinformatic and experimental approaches in translating target identification into a predictive and translationally robust antibiotic discovery platform (Cardona et al., 2025) (Table 1).

**TABLE 1:** Classes of antibiotic resistance targets and computational strategies used for their identification.

Target identification strategy	Essentiality mapping	Metabolic chokepoint analysis	Protein-protein interaction (PPI) networks	Synthetic lethality approaches	Virulence factor targeting	Resistance determinant targeting	Regulatory protein targeting	Host-pathogen interaction nodes
Core Biological Principle	Identifies genes indispensable for bacterial survival under normal or host-relevant conditions	Identifies enzymes uniquely responsible for production or consumption of essential metabolites	Identifies highly connected hub proteins essential for pathway coordination and network stability	Identifies non-essential gene pairs whose simultaneous inhibition causes lethality	Targets genes required for pathogenicity but not bacterial survival	Targets genes directly responsible for antibiotic resistance	Targets master regulators controlling resistance and virulence pathways	Targets bacterial survival mechanisms within host environments
Typical Representative Targets	DNA gyrase, RNA polymerase, PBPs, ribosomal proteins, MurA, FabI	Folate biosynthesis enzymes, lipid synthesis enzymes, DHFR, Mur pathway enzymes	Regulatory hubs, scaffold proteins, replication complexes, ribosomal assembly proteins	Parallel metabolic pathways, compensatory enzymes, redundant survival pathways	Type III secretion systems, toxins, adhesins, quorum sensing proteins	β-lactamases, efflux pumps, modifying enzymes, porins	Two-component systems, transcription factors, global regulators	Host receptor–pathogen ligand interactions, immune evasion proteins
Common Computational Methods	Tn-seq, CRISPRi screens, knockout libraries, comparative genomics	Genome-scale metabolic models (GEMs), Flux Balance Analysis (FBA), chokepoint analysis	PPI network reconstruction, centrality analysis, interactome modeling	Genetic interaction mapping, CRISPRi combinatorial screens, ML interaction prediction	Comparative genomics, virulence databases, host-pathogen interaction models	Resistome analysis, pan-genome analysis, comparative genomics	Regulatory network reconstruction, transcriptomics integration	Dual RNA-seq, systems biology, host-pathogen interaction modeling
Experimental Validation Methods	Gene knockout studies, MIC testing, growth inhibition assays	Enzyme inhibition assays, metabolic flux validation	Co-IP, pull-down assays, network perturbation studies	Double knockout validation, synergy testing	Infection models, virulence attenuation studies	Enzyme inhibition assays, susceptibility restoration studies	Reporter assays, transcriptomic validation	Infection models, host-cell assays
Translational Robustness	Very high because inhibition produces strong bactericidal effects and clear phenotypes	High due to systemic metabolic collapse after inhibition	Moderate; pathway collapse possible but validation is complex	Moderate to high, especially for validated combinations	Moderate because effects depend on infection context	Very high because inhibition restores antibiotic efficacy	Moderate	Moderate
Resistance Resilience	Moderate; strong selective pressure may accelerate resistance via mutations or bypass mechanisms	High because bottlenecks often have low redundancy and limited bypass potential	High because multiple downstream pathways are affected simultaneously	Very high because simultaneous escape from dual targets is difficult	Very high because selective pressure is reduced	High because therapy is adjunctive and resistance-modulating	High due to broad phenotypic control	Very high because targets are context-specific
Broad-Spectrum vs. Narrow-Spectrum Suitability	Highly suitable for broad-spectrum antibiotics, especially when conserved core genes are targeted	Suitable for both broad-spectrum and selective targeting, depending on pathway conservation	More suitable for narrow-spectrum and precision therapy	Particularly suitable for narrow-spectrum and combination therapy approaches	More suitable for narrow-spectrum precision therapy	Useful for both broad-spectrum restoration and targeted therapy	More suitable for narrow-spectrum and adaptive control strategies	Highly suitable for precision and adjunctive therapies
Clinical Relevance	Very high for classical antibiotic development	High for resistance-resilient drug development	Moderate to high for anti-virulence and systems targeting	Very high for resistance management strategies	High for anti-virulence therapeutics	Very high in resistant infections	Moderate to high	High for chronic and intracellular infections
Druggability Potential	High when active sites are structurally accessible	High if enzymes possess accessible catalytic pockets	Often limited due to shallow or dynamic interfaces	Moderate depending on paired target tractability	Moderate	High for enzymes, moderate for transporters	Moderate	Variable
Major Advantages	Strong biological relevance, validated targets, robust phenotypic outcomes	High systemic impact, reduced compensation, better pathogen selectivity	Systems-level targeting, anti-virulence potential, pathway-wide disruption	Enables combination therapy, reduces resistance emergence, expands target space	Reduced resistance pressure, preserves microbiota, strong host relevance	Restores activity of existing antibiotics, synergistic therapeutic effect	Broad phenotypic suppression, indirect resistance control	Enables host-directed therapy and adjunctive treatment
Key Limitations	Host toxicity risk due to homologous human targets; rapid resistance selection	Context-dependent metabolism; requires accurate metabolic reconstruction	Poor structural druggability; difficult validation	Experimentally complex, context-specific, difficult clinical translation	Limited immediate bactericidal effect	Resistance mechanism diversity and rapid evolution	Indirect effects may be difficult to predict	High biological complexity and host variability
Resistance Escape Risk	High	Low to moderate	Low	Very low	Very low	Moderate	Low	Very low
Selectivity Toward Pathogen	Moderate to high depending on ortholog filtering	High due to pathway specificity	High	High	Very high	High	High	Very high
Suitability for Combination Therapy	Moderate	High	Very high	Very high	Very high	Very high	High	Very high
Role in Precision Medicine	Moderate	High	Very high	Very high	Very high	Moderate	Very high	Extremely high
Typical Associated Databases/Tools	DEG, KEIO collection, TnCentral, CRISPR databases	KEGG, BioCyc, ModelSEED, COBRA Toolbox	STRING, BioGRID, Cytoscape, IntAct	SynLethDB, CRISPR screens, genetic interaction maps	VFDB, Victors, PHI-base	CARD, ResFinder, ARG-ANNOT	RegulonDB, RNA-seq integration platforms	HPIDB, dual RNA-seq platforms
Example Successful Applications	Fluoroquinolones targeting DNA gyrase; β-lactams targeting PBPs	Sulfonamides targeting folate pathway enzymes	Anti-virulence inhibitors targeting secretion system regulators	Combination therapies targeting folate and cell wall pathways	Quorum sensing inhibitors in *Pseudomonas aeruginosa*	β-lactamase inhibitors like clavulanate; efflux pump inhibitors	Histidine kinase inhibitors	Host-directed TB therapies

## 4 Structural bioinformatics for antibiotic target modeling and druggability assessment

Structural bioinformatics is a crucial area in modern antibiotic research, providing atomistic insight into bacterial targets that enable the rational assessment of druggability, selectivity, and mechanism of action. However, as AMR increasingly arises from complex structural adaptations, such as point mutations that alter binding pockets, conformational changes that reduce drug binding affinity, or the formation of multimeric complexes that protect catalytic sites, the need for structural approaches to translate genomic targets into viable chemical leads has become paramount (Seoane and Bou, 2021; Wu et al., 2025).

### 4.1 Experimental and predicted protein structures

**FIGURE 3:**
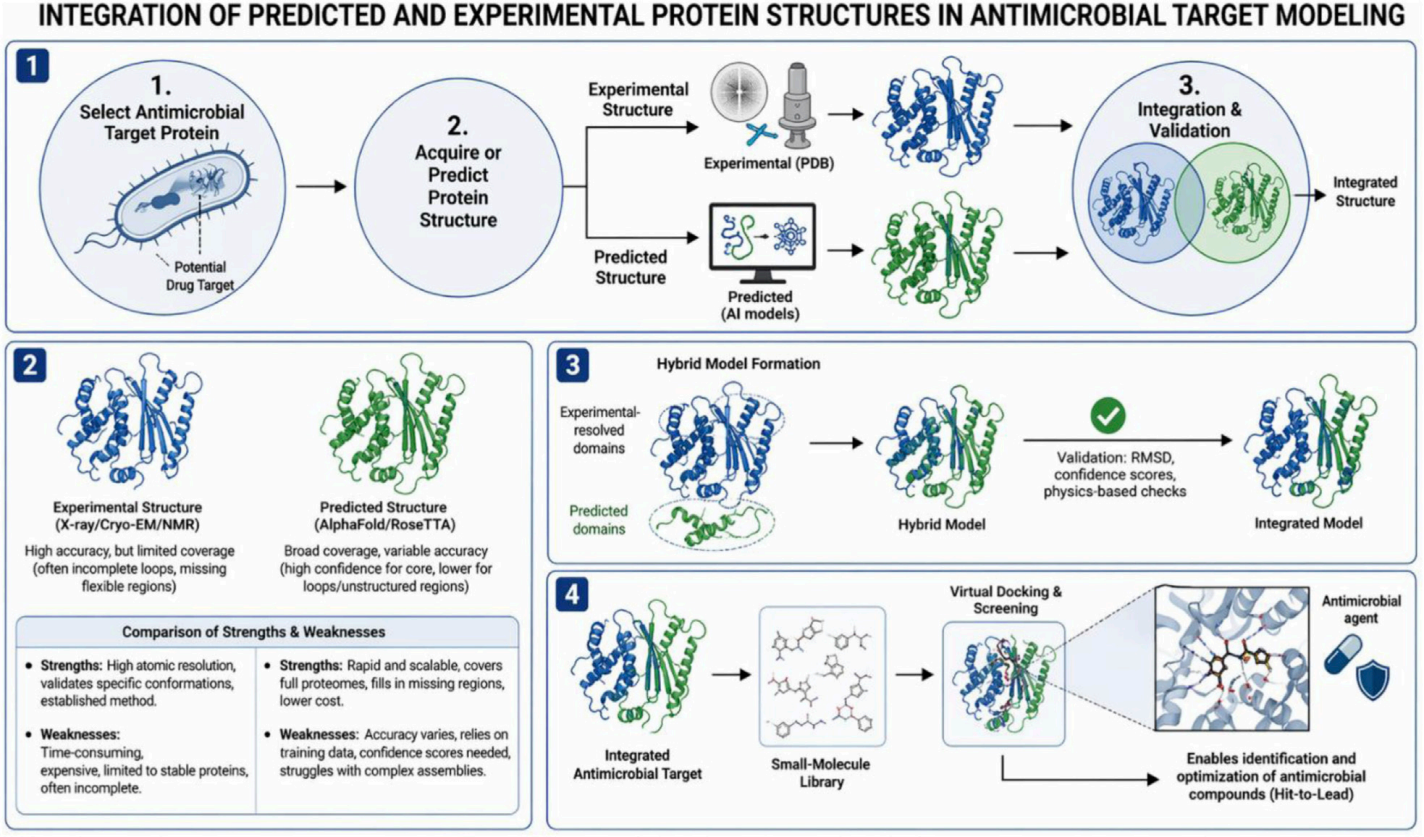
Workflow illustrating the integration of experimental and predicted protein structures for antimicrobial target modeling. Candidate target proteins are first selected, followed by structural acquisition from experimental methods such as X-ray crystallography, cryo-EM, or NMR, or from AI-based prediction tools including AlphaFold and Rosetta. Experimental structures provide high accuracy, while predicted models offer broader coverage and fill unresolved regions. These datasets are combined to generate hybrid structural models and validated using RMSD, confidence scores, and physics-based assessments. The validated integrated structures are then used for virtual docking and screening of small-molecule libraries to identify promising antimicrobial lead compounds.

Historically, experimental approaches in structural biology, specifically X-ray crystallography, nuclear magnetic resonance (NMR) spectroscopy, and cryo-EM, have been the foundation for structure-based approaches to antibiotic discovery. X-ray crystallography remains the most popular approach for bacterial enzymes and soluble proteins, providing high-resolution structures amenable to docking and structure-based optimization (Maveyraud and Lionel, 2020; Rubach et al., 2025). NMR spectroscopy has provided important information on protein dynamics, ligand binding, and transient conformational states, particularly for smaller bacterial proteins and regulatory regions. More recently, improvements in cryo-EM have revolutionized the field by allowing near-atomic resolution structures of large, flexible, and multimeric bacterial complexes that were previously inaccessible, including ribosomes, secretion systems, efflux pumps, and membrane-associated machineries that play pivotal roles in antibiotic resistance (Kang, 2019; Puthenveetil and Vinogradova, 2019; Son et al., 2024). Despite these advances, experimental structure determination remains a time- and resource-intensive process, and a large number of clinically relevant bacterial proteins remain without experimentally determined structures. AI-based prediction algorithms have significantly alleviated this challenge by the advent of AI-based structure prediction algorithms, specifically AlphaFold2 (https://alphafoldserver.com/welcome) and RoseTTAFold (https://github.com/RosettaCommons/RoseTTAFold). These tools have shown unprecedented accuracy in predicting protein folds directly from amino acid sequences, often approaching experimental resolution for globular domains (Baek et al., 2021; Qiu et al., 2024; Wang J. et al., 2024). In the context of AMR research, predicted structures have provided structural insight into resistance determinants, essential enzymes, transcriptional regulators, and hypothetical proteins in a wide range of pathogens, thus enabling structure-based analyses at the proteome scale. Notably, predicted structures are not simply surrogates for experimental data but rather hypothesis-generating tools. They allow for rapid evaluation of fold conservation, identification of catalytic motifs, prediction of ligand-binding pockets, and comparison of resistance-associated mutations (Ko et al., 2023; Süssmuth et al., 2025). Combined approaches that merge predicted models with low-resolution experimental data or mutational constraints are becoming increasingly common, particularly for resistance targets. However, validation and confidence estimation are still crucial, particularly in regions of low confidence or predicted disorder (Figure 3).

### 4.2 Binding site and pocket identification

With a reliable protein structure, either experimental or predicted by computation, in hand, the task of ligand-binding site prediction becomes a critical component of druggability analysis. Binding site prediction algorithms can be broadly classified into geometry-based and energy-based prediction tools, each with its own set of advantages. Geometry-based prediction algorithms rely on the identification of pockets, clefts, or cavities based on geometric properties such as volume, depth, enclosure, and surface properties (Liao et al., 2022; Pliushcheuskaya and Künze, 2025). These algorithms are computationally fast and amenable to high-throughput screening of large target portfolios, making them highly useful in early-stage antibiotic target selection. In contrast, energy-based prediction algorithms assess the physicochemical desirability of ligand binding based on the calculation of interaction energy between probe molecules and the protein surface (Carracedo-Reboredo et al., 2021; Ahmad et al., 2026; Grosjean and Biggin, 2026). These algorithms predict hydrophobic hotspots, hydrogen bond donors and acceptors, and electrostatic complementarity, often providing a more refined view of ligand ability. In the context of antibiotic discovery, where bacterial proteins often display shallow or atypical binding pockets, energy-based prediction algorithms have been shown to predict cryptic binding sites that are not detectable by geometric analysis alone (Ramamoorthy et al., 2013; Damm-Ganamet et al., 2019; Zhao et al., 2020). In addition to traditional active sites, there has been a growing focus on allosteric binding sites. Allosteric inhibition is a highly effective approach to resistance, where regulatory or conformational control points are targeted instead of conserved catalytic residues. Structural bioinformatics algorithms have increasingly incorporated conformational sampling, normal mode analysis, and molecular dynamics-derived ensembles to predict transient allosteric pockets that form during protein motion. Several successful antibacterial agents have been shown to target noncanonical sites, emphasizing the need to extend pocket prediction algorithms beyond static structures (Butler et al., 2024; Sanapalli et al., 2026a; Gao et al., 2026) (Figure 3).

### 4.3 Challenges in modeling bacterial targets

Despite progress, modeling bacterial proteins remains a unique challenge that impedes structure-based antibiotic discovery. The first challenge is conformational flexibility. Many bacterial proteins, particularly those involved in metabolism, transcription, or signaling, exhibit large conformational transitions upon ligand binding, substrate turnover, or complex formation (Zhang et al., 2021). This makes it difficult for static structures, whether experimental or modelled computationally, to accurately represent a single conformational state, which may not even be representative of biologically relevant, binding-competent conformations. This issue is especially true for resistance mutations, which can cause subtle changes in conformational equilibria rather than simply blocking binding sites (Marco and Gago, 2007). Therefore, the integration of molecular dynamics simulations with ensemble docking approaches has become increasingly valuable for modeling functional flexibility. The second challenge arises from modeling multimeric protein complexes. Many of the most important antibacterial targets, such as ribosomes, replisomes, and cell wall biosynthesis complexes, function as large multimeric assemblies rather than as individual proteins. The prediction of protein interfaces, stoichiometry, and cooperative conformational changes in these assemblies remains challenging, particularly when subunit composition is variable across species or in response to environmental conditions (Moroy et al., 2015; Harmalkar and Gray, 2021; Yuce et al., 2024). While cryo-EM has eased some of the experimental difficulties, computational modeling of multimeric bacterial protein complexes remains a challenge that requires a careful combination of structural prediction, co-evolutionary data, and experimental constraints. Further, membrane proteins represent another category of bacterial targets, which are even more challenging (Corum et al., 2024; Varadi et al., 2025; Lu et al., 2025). Efflux pumps, transporters, and membrane-associated enzymes are key to AMR, either by reducing intracellular drug concentrations or by altering drug targets. Their hydrophobic nature, dynamic structure, and membrane dependence make experimental structure determination and modeling even more challenging (Gaurav et al., 2023). Although AI-based prediction tools have significantly improved the availability of membrane protein structures, accurate modeling of binding sites embedded in membranes and drug permeation pathways often requires simulations of the membrane and customized force fields. Finally, selectivity is a concern in modeling bacterial targets (Leman et al., 2015; Jawarkar et al., 2026). Many essential bacterial proteins have structural homologs in human proteins, which can lead to off-target toxicity. This issue can be addressed by structural bioinformatics, which uses comparative modeling and orthology-informed analyses to identify pathogen-specific pockets, distinct surface regions, or unique conformational states that are absent in the human homologs (Khan et al., 2022; Azevedo et al., 2024). The inclusion of selectivity filters early in the pipeline can prevent late-stage failures and improve translational potential (Figure 3).

## 5 Virtual screening and molecular docking pipelines for antibiotic resistance

**FIGURE 4:**
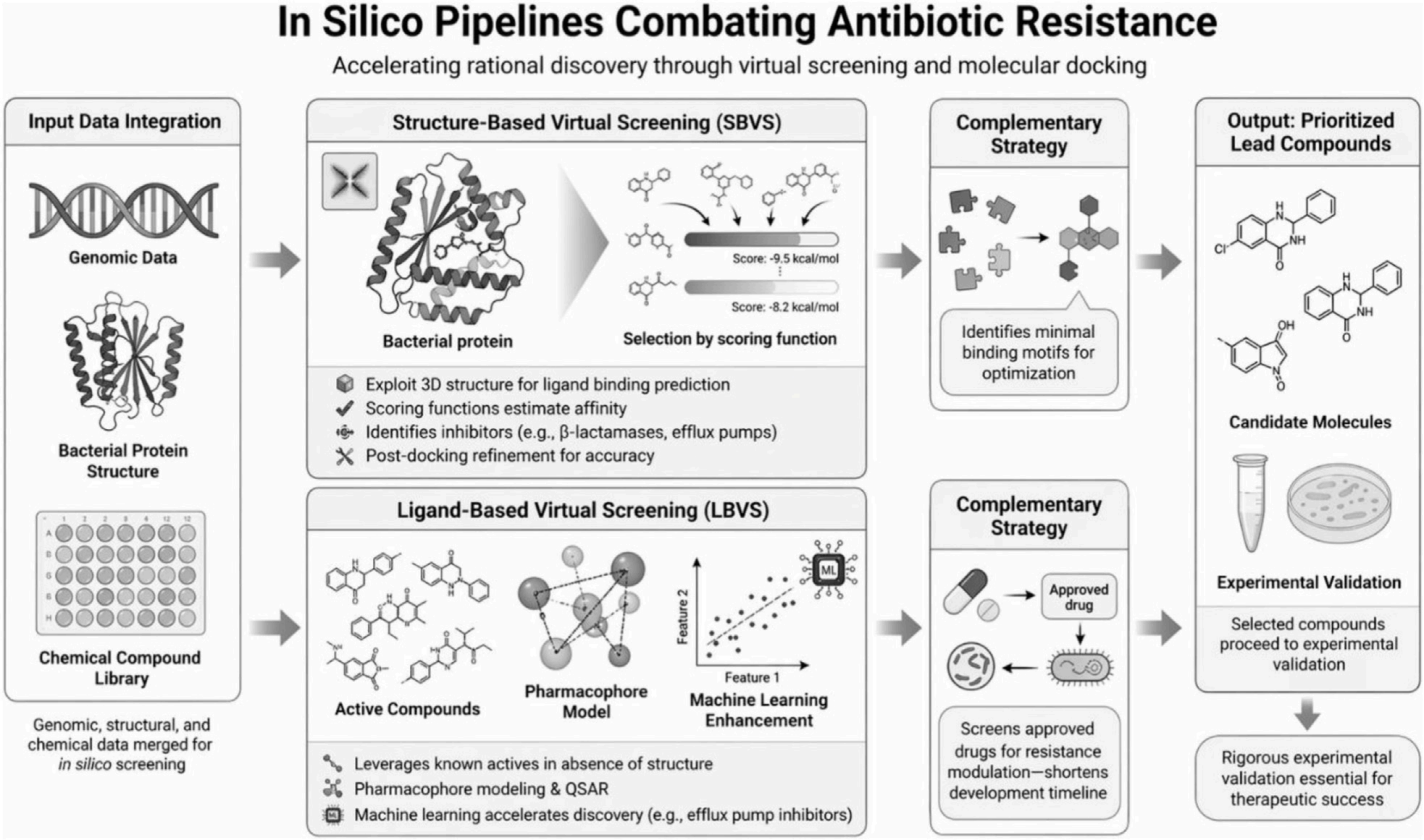
Schematic representation of *in silico* pipelines used to combat antibiotic resistance through computational drug discovery. The workflow begins with input data integration, combining genomic information, bacterial protein structures, and chemical compound libraries. Structure-based virtual screening (SBVS) utilizes three-dimensional protein structures and molecular docking to predict ligand binding and rank compounds based on scoring functions, enabling identification of inhibitors targeting key bacterial proteins such as β-lactamases and efflux pumps. LBVS complements this approach by using known active compounds, pharmacophore modeling, QSAR analysis, and machine learning to predict new candidate molecules when structural data are limited. Additional complementary strategies include fragment-based optimization and drug repurposing using approved drugs. Promising molecules are prioritized as lead compounds and subsequently subjected to rigorous experimental validation to confirm antimicrobial activity and therapeutic potential.

Virtual screening and molecular docking constitute core components of modern *in silico* drug discovery pipelines targeting antibiotic resistance. These computational approaches enable systematic prioritization of candidate compounds from vast chemical spaces by predicting their likelihood of interacting with bacterial targets implicated in resistance mechanisms. When integrated with genomic and structural data, virtual screening strategies accelerate lead identification, reduce experimental costs, and support rational antibiotic discovery in an era of escalating AMR (Aarón et al., 2025; Bess et al., 2026) (Figure 4) (Table 2).

**TABLE 2:** Major tools and databases used in virtual screening and molecular docking frameworks for antibiotic discovery.

Category	Tool/Database	Primary function	Application in antibiotic discovery	Significance	References
Protein Structure Database	Protein Data Bank (PDB)	Repository of experimentally resolved 3D protein structures	Provides crystal/cryo-EM structures of bacterial targets such as β-lactamases, PBPs, and efflux pumps	Foundation for structure-based virtual screening (SBVS)	Berman et al. (2000)
AI-Based Structure Prediction	AlphaFold	Predicts high-accuracy protein structures from amino acid sequences	Enables modeling of unresolved resistance proteins and novel bacterial targets	Expands target accessibility beyond experimentally solved proteins	Jumper et al. (2021)
Binding Pocket Prediction	DoGSiteScorer	Identification of ligand-binding pockets and druggability assessment	Detects catalytic and allosteric pockets for docking	Improves target prioritization	Volkamer et al. (2012)
Binding Hotspot Mapping	FTMap	Identifies binding hotspots using probe mapping	Detects cryptic and druggable sites in bacterial proteins	Supports rational lead optimization	Kozakov et al. (2015)
Molecular Docking	AutoDock/AutoDock Vina	Predicts ligand binding poses and affinity	Screening inhibitors against β-lactamases, efflux pumps, DNA gyrase	Widely used open-source docking platform	Trott and Olson, (2010)
Molecular Docking	Glide (Schrödinger)	High-precision molecular docking and scoring	Advanced docking of lead candidates with XP precision	High docking accuracy for hit prioritization	Halgren et al. (2004)
Molecular Docking	GOLD	Genetic algorithm-based docking	Flexible docking for difficult targets and metalloenzymes	Useful for complex active-site interactions	Verdonk et al. (2003)
Chemical Database	ZINC Database	Commercially available chemical compound library	Virtual screening of millions of drug-like molecules	Large-scale ligand discovery	Irwin and Shoichet, (2005)
Chemical Database	PubChem	Bioactive small molecules and compounds	Compound retrieval and antibacterial candidate screening	Broad chemical diversity	Kim (2021)
Drug Repurposing Database	DrugBank	Approved drugs and pharmacological information	Identification of repurposable non-antibiotic compounds	Accelerates translational development	Wishart et al. (2018)
Pharmacophore Modeling	Phase (Schrödinger)	Ligand-based pharmacophore generation	Discovery of efflux pump inhibitors and scaffold hopping	Useful when structural data are limited	Dixon et al. (2006)
QSAR Modeling	MOE	Descriptor generation and QSAR development	Predicts antibacterial activity using molecular descriptors	Supports LBVS	Vilar et al. (2008)
Molecular Dynamics	GROMACS	Simulation of protein-ligand stability	Validates docking poses and binding persistence	Reduces false positives after docking	Pronk et al. (2013)
ADMET Prediction	SwissADME	Predicts absorption and drug-likeness	Early lead optimization for permeability and toxicity	Improves clinical translatability	Daina et al. (2017)
Toxicity Prediction	pkCSM	Toxicity and pharmacokinetic prediction	Eliminates compounds with poor safety profiles	Reduces late-stage failures	Pires et al. (2015)

### 5.1 Structure-based virtual screening (SBVS) of antibiotic resistance determinants

SBVS is a computational strategy that leverages three-dimensional structural data of bacterial target proteins to predict the binding modes of ligands and to estimate their relative binding affinities. The recent explosion in the number of protein structures solved by X-ray crystallography and cryo-electron microscopy, coupled with advances in homology and comparative modeling, has led to an increased availability of high-confidence structural templates for essential bacterial enzymes, resistance determinants, and virulence-associated proteins (Zhu H. et al., 2022; Han et al., 2023). The increased availability of structural templates has enabled large-scale implementation of SBVS, facilitating the systematic exploration of large chemical libraries against biologically relevant bacterial targets (Fleeman et al., 2015). Further, molecular docking is the core computational step in SBVS and is typically carried out using algorithms, such as AutoDock (https://autodock.scripps.edu/), Glide (https://www.glideapps.com/), and GOLD (https://www.ccdc.cam.ac.uk/solutions/software/gold/). These tools model ligand-protein recognition events by sampling ligand conformations and poses within pre-defined binding sites, followed by the calculation of intermolecular interactions (Ferreira et al., 2015; Agu et al., 2023; Mursal et al., 2024). Additionally, binding poses are scored using physicochemical parameters that include hydrogen-bonding networks, hydrophobic interactions, electrostatic complementarity, and van der Waals interactions. To better model the inherent flexibility of bacterial proteins, docking protocols often include flexible ligand sampling and, in some cases, limited side-chain flexibility of the receptor protein, thus improving pose prediction for proteins that display conformational flexibility (Patil et al., 2010; Singh et al., 2011; Blanes-Mira et al., 2022; Ma et al., 2024; Meghana and Sangi, 2026). Moreover, scoring functions are a key component of SBVS, where they are used to rank docked molecules based on their predicted binding affinity. Traditional scoring functions, including empirical, force field-based, and knowledge-based scoring functions, are characterized by different underlying assumptions and trade-offs. Although these scoring functions enable high-throughput screening of candidate molecules, their accuracy is compromised by simplified models of energetics, especially in cases where the receptor exhibits high flexibility, metal coordination, or water-mediated interactions (Guedes et al., 2018; Meli et al., 2022; Pellicani et al., 2023; Angelova et al., 2025). This results in a high incidence of false-positive and false-negative predictions, which can be addressed by post-docking refinement methods such as consensus scoring, rescoring with more physics-based models, or molecular dynamics-based validation (Yang et al., 2005; Rastelli and Pinzi, 2019). Additionally, one of the most important but frequently underestimated limitations of current *in silico* antibiotic discovery pipelines is the prediction of compound permeability across the Gram-negative bacterial outer membrane. Unlike Gram-positive organisms, Gram-negative pathogens possess an additional lipopolysaccharide-rich outer membrane that creates a highly selective permeability barrier, substantially limiting intracellular access of many otherwise potent antibacterial compounds (Ferreira and Kasson, 2019; Nazir et al., 2025). Consequently, Strong target binding predicted by docking does not always translate into whole-cell antibacterial activity because permeability and efflux strongly influence intracellular drug accumulation. Additionally, current models often underrepresent porin uptake, physicochemical constraints, and AcrAB-TolC efflux, while limited datasets and assay variability reduce prediction reliability (Ferreira and Kasson, 2019). Furthermore, permeability and efflux are often interdependent rather than independent variables, making simple descriptor-based prediction unreliable. Recent efforts integrating molecular dynamics simulations, permeability rules, ML classifiers, and structure-accumulation relationship models have improved prediction accuracy, but robust prospective validation remains limited (Milenkovic et al., 2024). Therefore, future computational antibiotic discovery pipelines must move beyond target-centric screening and incorporate permeability-aware design frameworks as a mandatory step, particularly for Gram-negative priority pathogens, such as *Acinetobacter baumannii*, *Pseudomonas aeruginosa*, and carbapenem-resistant *Enterobacterales*.

Despite the above limitations, SBVS has shown its potential in the discovery of inhibitors that target bacterial antibiotic resistance mechanisms. Structure-based docking experiments have successfully identified small-molecule inhibitors of resistance-conferring proteins, such as β-lactamases, multidrug efflux pumps, and essential metabolic enzymes (Spyrakis et al., 2017; Zhu H. et al., 2022). Significantly, SBVS allows for the rational exploitation of conserved structural elements and catalytic topologies, which can be used to design molecules that are active against resistant bacterial strains. However, many virtual screening efforts have been conducted against crystallographically characterized metallo-β-lactamases, including variants of NDM-1 and VIM, and have yielded candidate inhibitors that bind to conserved residues in the active site (Spyrakis et al., 2015; Spyrakis et al., 2017). Follow-up biochemical studies and bacterial susceptibility testing have confirmed enzymatic inhibition and a return of β-lactam susceptibility in resistant isolates (Jendrzejewska and Karwowska, 2022). In aggregate, these findings highlight the value of SBVS as a hypothesis-generating and prioritization tool when used in the context of well-characterized bacterial resistance targets.

### 5.2 Harnessing pharmacophore and QSAR models to overcome antibiotic resistance

LBVS is applied when there is no or limited structural information available for the target protein. Unlike SBVS, LBVS relies on information extracted from known active compounds to discover new molecules that share similar physicochemical or pharmacophoric properties. Pharmacophore modeling is a core component of LBVS, which encompasses the primary molecular characteristics necessary for biological activity, such as hydrogen bond donors or acceptors, aromatic rings, hydrophobic areas, and charged groups (Vázquez et al., 2020; Sciabola et al., 2022). Pharmacophore models can be deduced from a collection of known antibiotics or resistance inhibitors and can then be used to screen compound libraries for molecules that fit the specified spatial and chemical requirements. This enables efficient identification of structurally diverse compounds that preserve key characteristics responsible for biological activity (Ananna et al., 2024). Moreover, quantitative structure-activity relationship (QSAR) modeling is another powerful LBVS technique. QSAR models describe mathematical correlations between molecular descriptors, such as molecular weight, lipophilicity, electronic properties, and topological indices, and biological activity (Bastikar et al., 2022). Recent studies have further demonstrated the translational relevance of ligand-based screening and network pharmacology in identifying anti-virulence therapeutics against multidrug-resistant pathogens. For example, a study on marine-derived *Bacillus safensis* DJ1 metabolites showed significant inhibitory effects against *P. aeruginosa* virulence through an integrated experimental and computational framework. Using network pharmacology, molecular docking, and molecular dynamics simulations, the authors identified key interactions between bacterial secondary metabolites and major virulence-associated targets involved in quorum sensing, biofilm formation, and pathogenic signaling pathways (Kachhadiya et al., 2026). Further, ML and statistical models, such as random forests (RF), support vector machines (SVM), and deep neural networks (DNN), have greatly improved QSAR model performance by detecting non-linear relationships in complex data sets. In the battle against antibiotic resistance, LBVS has been shown to be helpful in identifying novel inhibitors of recalcitrant enzymes and resistance mechanisms by leveraging insights from known antibiotic structures (Mohseni and Ghorbani, 2024; Bagdad and Miteva, 2024). For instance, pharmacophore modeling and QSAR-driven screening have enabled the identification of efflux pump inhibitors. These agents do not kill bacteria *per se* but increase the intracellular accumulation of antibiotics. By employing known efflux inhibitors as training examples, ligand-based models have identified chemically distinct molecules that can inhibit the prominent efflux pumps, thereby re-sensitizing multidrug-resistant bacteria to fluoroquinolones, tetracyclines, and β-lactam antibiotics (Stermitz et al., 2000; Lomovskaya et al., 2001; Nargotra et al., 2009; Brincat et al., 2011). These results demonstrate the utility of LBVS in situations where high-resolution target structures are not available or when resistance is mediated by complex, multi-faceted mechanisms. However, there are also some inherent limitations to LBVS approaches, which are dependent on the quality and representativeness of the training sets, and which can fail to detect activity in molecules acting via novel mechanisms that are not captured by known ligands (Gimeno et al., 2019; Elsaka et al., 2026).

### 5.3 Fragment-based drug design (FBDD) for resistance-resilient antibiotics

FBDD is an alternative approach that combines computational and experimental approaches to discover low-molecular-weight chemical fragments that can specifically bind to biologically relevant target sites with modest affinity but high ligand efficiency. Unlike traditional high-throughput screening approaches, which are biased toward large and complex molecules, FBDD is based on the idea of using minimal chemical scaffolds that can maximize binding interactions per atom, thus offering efficient leads for subsequent optimization (Li, 2020; Kirkman et al., 2024). Fragment libraries are generally small, chemically diverse compounds that are designed to probe a wide range of chemical space within a small molecular set. Computational screening of these libraries enables the systematic discovery of minimal binding motifs that can specifically bind to conserved regions of bacterial targets, such as the catalytic active site and regulatory or allosteric pockets that are involved in antibiotic resistance (Casu et al., 2017; Konaklieva and Plotkin, 2023; AlKharboush et al., 2025). Because of their small size and simplicity, fragments can potentially bind to sites that are sterically occluded to larger molecules. After the discovery of fragment hits, structure-based optimization approaches are used to improve binding affinity, selectivity, and pharmacological properties. Fragment growing is the stepwise expansion of the initial scaffold through rational functional group addition to enhance binding pocket interactions (Zender et al., 2020; Kaya et al., 2022; Mielniczuk et al., 2024; Walsh et al., 2025). Conversely, fragment linking aims to chemically merge two or more fragments that bind to adjacent regions, creating a single molecule with higher binding affinity and cooperativity. Molecular docking and molecular dynamics simulations are key components of these methods, predicting binding orientations, evaluating binding interactions, and simulating the energetic effects of chemical modifications (Neto et al., 2020; Pandya et al., 2024). Moreover, FBDD is highly desirable in antibiotic discovery because it enables the targeting of evolutionarily conserved bacterial attributes while maintaining chemical diversity, thus lowering the chances of cross-resistance with existing antibiotics. Computer-assisted fragment screening against essential bacterial enzymes has successfully identified high ligand-efficiency fragments that interact with conserved active or allosteric sites. Structure-based optimization, guided by docking and dynamics simulations, has led to the identification of compounds with higher binding affinity and measurable antibacterial activity (Arif et al., 2022; Konaklieva and Plotkin, 2023; Breijyeh and Karaman, 2023). Biochemical and microbiological assays have validated the translational potential of fragment-derived compounds. However, despite these benefits, fragment hits usually need a lot of optimizations to reach potentially useful potency. As such, there is a need for a combination of predictions and experimental validation.

### 5.4 Repositioning approved drugs to combat antibiotic resistance

Drug repurposing involves the use of already approved or clinically evaluated drugs to discover new, yet unforeseen, antibacterial, or resistance-modulating properties. Considering the difficulties involved in discovering new antibiotics, specifically high failure rates, increasing costs, and prolonged development times, *in silico* drug repurposing has been recognized as a highly effective complementary approach to combat antimicrobial resistance (Khzem and Wali, 2025). By leveraging on compounds that have already been characterized for their pharmacokinetic, safety, and toxicity profiles, drug repurposing significantly shortens the time and risk involved in the clinical development of new drugs. Computer-assisted screening of approved drug libraries allows for the rapid identification of compounds that can potentially inhibit bacterial resistance determinants, improve the efficacy of existing antibiotics, or target essential survival pathways (Aloni-Grinstein et al., 2025; Tiwana et al., 2025; Guo et al., 2026). *In silico* screening strategies have successfully identified repurposed candidates that can inhibit efflux pump expression, interfere with biofilm development, or re-sensitize bacteria to existing first-line antibiotics. Such properties can be realized at sub-inhibitory concentrations when repurposed drugs are used in combination therapy regimens, emphasizing their potential as resistance modulators rather than antimicrobial agents (Liu et al., 2021; Migliaccio et al., 2024; Aloni-Grinstein et al., 2025; Szemerédi et al., 2026). Moreover, network-based and systems pharmacology approaches support repurposing approaches by combining information on host-pathogen interactions and bacterial stress response networks. These studies have identified compounds that indirectly reduce bacterial fitness by modulating host immune responses or by disrupting bacterial adaptive responses (Saini et al., 2023; Banerjee, 2025). Notably, repurposed compounds often have synergistic effects when combined with traditional antibiotics, allowing for the potential to overcome existing resistance mechanisms. One of the most successful examples of the utility of computational repurposing is the discovery of halicin using AI-driven virtual screening approaches (Cantrell et al., 2022; Cunha-Ferreira et al., 2025). Originally designed as a c-Jun N-terminal kinase inhibitor, halicin was computationally predicted to have antibacterial properties and was later demonstrated to have potent activity against multidrug-resistant bacteria, including *Acinetobacter baumannii*, *in vitro* and *in vivo* infection models (Stokes et al., 2020; Booq et al., 2021; El et al., 2025). Further repurposing studies have also identified non-antibiotic compounds that can inhibit efflux pumps, disrupt biofilms, or increase antibiotic penetration, further validating the utility of this approach. However, despite these benefits, repurposing of drugs faces some challenges, such as suboptimal antibacterial activity, pharmacodynamic limitations in infection sites, and intellectual property issues. In addition, repurposed drugs may exert selective pressures that contribute to the development of alternative resistance mechanisms (Kincses et al., 2025; Tiwana et al., 2025; Momin-Reza et al., 2025). Therefore, any predictions made by *in silico* models must be validated rigorously by experimental and clinical studies.

### 5.5 Structure-guided virtual screening and molecular docking frameworks for antibiotic discovery: methodology, applications, and translational significance

Virtual screening and molecular docking have become central components of modern antibiotic discovery pipelines because they enable the rapid identification, prioritization, and optimization of candidate compounds against bacterial resistance targets before costly experimental validation (Noumi et al., 2025). These approaches are particularly important in addressing AMR, where conventional antibiotic discovery strategies are often associated with long development timelines, high financial burden, and substantial late-stage failure rates. By integrating structural biology, computational chemistry, and pharmacological prediction, virtual screening provides a rational and efficient route for discovering new antimicrobial agents (Wu et al., 2025). The process typically begins with the identification of biologically relevant bacterial targets involved in resistance or survival, such as β-lactamases, efflux pumps, DNA gyrase, topoisomerases, penicillin-binding proteins, and virulence-associated enzymes (Belay et al., 2024). Further, high-resolution structural information is obtained either from experimentally resolved structures available in the PDB or from AI-based prediction platforms, such as AlphaFold. For example, crystallographic studies of New Delhi metallo-β-lactamase-1 (NDM-1), one of the most clinically important carbapenem resistance enzymes, enabled the identification of conserved catalytic zinc-binding pockets that became major docking targets for inhibitor design (King and Strynadka, 2011; Vasudevan et al., 2022). Similarly, structural characterization of AcrB efflux pumps in Gram-negative bacteria has facilitated the screening of efflux pump inhibitors capable of restoring antibiotic susceptibility (Bhowmik et al., 2026). Additionally, binding pocket prediction tools, such as DoGSiteScorer and FTMap are then used to identify active sites, allosteric pockets, and cryptic druggable hotspots. Once target sites are defined, large chemical libraries from ZINC, PubChem, and DrugBank are screened using molecular docking platforms, such as AutoDock, Glide, and GOLD (MacKerell et al., 2020). These tools predict ligand-binding conformations and estimate interaction energies based on hydrogen bonding, hydrophobic interactions, electrostatic complementarity, and van der Waals forces. In a notable study targeting NDM-1, structure-based virtual screening of thousands of compounds identified aspergillomarasmine A as a potent inhibitor capable of restoring meropenem sensitivity in resistant Enterobacteriaceae. Subsequent biochemical assays confirmed inhibition of metallo-β-lactamase activity, while murine infection models demonstrated significant therapeutic efficacy, providing strong translational validation. Moreover, post-docking refinement is essential to reduce false positives and improve prediction accuracy (King et al., 2014; Rotondo et al., 2020). A major strength of modern *in silico* antibiotic discovery lies not only in primary computational screening but also in the iterative integration of experimental feedback that progressively improves enrichment efficiency during secondary and tertiary selection stages. Initial virtual screening campaigns often begin with millions of compounds, which are first reduced to thousands of high-scoring candidates based on docking scores, pharmacophore matching, or machine learning predictions (Sierra-Hernandez et al., 2025). However, these early-stage selections typically contain substantial false-positive rates due to limitations in scoring functions, structural assumptions, and incomplete biological context. Secondary selection stages involving biochemical enzyme inhibition assays, minimum inhibitory concentration (MIC) testing, bacterial accumulation studies, and cytotoxicity profiling provide critical feedback that enables recalibration of docking parameters, rescoring models, and ML classifiers (Chen et al., 2020; Bender and Cortés-Ciriano, 2021). For example, in β-lactamase inhibitor discovery and halicin-like AI-guided screening workflows, iterative rounds of computational prediction followed by experimental validation substantially improved hit enrichment by eliminating non-permeable compounds, weak binders, and false-positive scaffold classes before lead optimization. A chronological filtering framework, such as progression from 10^6 screened compounds to 10^3 prioritized hits, followed by 10^2 experimentally validated candidates and finally fewer than 10 optimized leads more accurately reflect real translational workflows than single-step screening models (King et al., 2014; Stokes et al., 2020). This iterative feedback loop significantly reduces development costs, improves model generalizability, and increases the probability of identifying clinically viable antimicrobial candidates (Liu et al., 2026). Further, molecular dynamics simulations using GROMACS and rescoring strategies help assess binding stability under dynamic biological conditions. For example, molecular dynamics-assisted docking studies targeting DNA gyrase in methicillin-resistant *Staphylococcus aureus* (MRSA) successfully identified fluoroquinolone analogs with improved stability and binding persistence, which were later validated through *in vitro* antibacterial assays (Elseginy and Anwar, 2022). Similarly, docking combined with molecular dynamics has been used to optimize inhibitors of MurA and FabI, essential bacterial enzymes involved in cell wall and fatty acid biosynthesis (Parkhill and Johnson, 2024). Furthermore, ADMET prediction tools, such as SwissADME and pkCSM are subsequently employed to evaluate absorption, distribution, metabolism, excretion, toxicity, and permeability before experimental testing. This step significantly improves translational success by eliminating compounds with poor pharmacokinetic profiles or predicted toxicity (Orujova et al., 2025; Lahlou et al., 2026). A prominent example is the AI-assisted discovery of halicin, originally developed as a c-Jun N-terminal kinase inhibitor. Deep learning-guided virtual screening identified halicin as a potent antibacterial agent with broad-spectrum activity against multidrug-resistant pathogens, including *A. baumannii* and *Mycobacterium tuberculosis* (Wang S. et al., 2024). Experimental validation confirmed its novel mechanism of action involving disruption of proton motive force and demonstrated strong efficacy in mouse infection models. The significance of this framework lies in its ability to reduce millions of compounds to a small number of highly prioritized candidates for wet-lab validation (Stokes et al., 2020). It supports rational targeting of conserved resistance determinants, facilitates drug repurposing, improves hit-to-lead efficiency, and enables the development of resistance-resilient antibiotics. Thus, virtual screening and molecular docking serve as the critical bridge between genomic target identification and clinically relevant lead compound development, making them indispensable tools in contemporary *in silico* antibiotic discovery pipelines.

## 6 ML and AI-driven antibiotic discovery pipelines

ML and AI have emerged as key elements of contemporary *in silico* antibacterial discovery pipelines, revolutionizing the discovery, optimization, and evaluation of antibacterial agents in the context of increasing antimicrobial resistance. Unlike conventional virtual screening approaches, which are largely based on pre-defined physicochemical rules or fixed protein-ligand interactions, ML- and AI-based approaches rely on large and diverse datasets to identify complex, non-linear relationships between molecular structure, biological activity, and bacterial phenotype (Diéguez-Santana and González-Díaz, 2023; Lin et al., 2025; Sanapalli et al., 2026b). A variety of ML algorithms, such as RFs, SVMs, and deep learning models, have been extensively used to predict antimicrobial activity, classify compounds as potential actives or inactives, and rank candidates for experimental validation. These approaches combine diverse feature types, including molecular fingerprints, physicochemical properties, graph-based molecular representations, protein sequence embeddings, and genome-based functional annotations, to comprehensively model compound-target-organism interactions (Stokes et al., 2020; Cesaro et al., 2023b). Moreover, RFs and SVMs have been shown to be highly effective for early-stage antimicrobial predictions, owing to their robustness to noise and interpretability, while deep learning models are best suited for modeling high-dimensional, non-linear patterns that often underlie antibacterial activity (Yun et al., 2024; Bonifacio-Velez de Villa et al., 2025). One of the most appropriate examples of AI-assisted discovery is the discovery of halicin, which was discovered using deep learning-assisted screening of large chemical libraries trained on data related to bacterial inhibition of growth rather than traditional structural docking. This approach led to the discovery of a structurally atypical compound with broad-spectrum and potent activity against multidrug-resistant bacteria, including carbapenem-resistant bacteria, which was later experimentally validated to have efficacy in animal models of infection (Booq et al., 2021; Kao et al., 2024; Mulat et al., 2025). Crucially, halicin has a novel mechanism of action that is distinct from existing antibiotics, demonstrating that AI-assisted screening can potentially outperform traditional chemical insight and identify compounds that target known resistance mechanisms in a novel way. In addition to hit discovery, deep learning has also been used for *de novo* antibiotic design, representing a shift from predictive to generative AI in drug discovery (Zhang M. et al., 2024; Wang S. et al., 2024). Further, graph neural networks (GNNs), which are designed to directly process graph-structured data such as molecules, have been used for the design of novel compounds by learning representations of atoms and bonds that are contextually informed, allowing for the simultaneous optimization of antibacterial activity, drug-likeness, and toxicity-related properties. Concurrently, SMILES string-based generative models, such as recurrent neural networks, variational autoencoders, and transformer models have been shown to have the ability to generate valid and chemically diverse molecules conditioned on desired antimicrobial or pharmacokinetic properties (Besharatifard and Vafaee, 2024; Zhang O. et al., 2025; Boudza et al., 2026). Some of these AI-designed molecules have since shown *in vitro* antibacterial activity, offering proof-of-concept that generative AI can venture into unexplored areas of chemical space. Moreover, ML and AI have also been successfully used for drug repurposing, which is a highly appealing approach in antibiotic development due to the high cost and failure rate associated with *de novo* design (Gangwal and Lavecchia, 2024; Dharmasivam et al., 2026). By using virtual screening of libraries of approved and clinically investigated drugs, ML algorithms have identified compounds originally developed for non-infectious diseases that possess unexpected antibacterial or resistance-modulating properties, such as efflux pump inhibitors, biofilm disruptors, or synergistic potentiators of existing antibiotics. The key advantage of this approach for translation lies in the pre-existing knowledge of safety, pharmacokinetics, and toxicity profiles, which allows for rapid translation from *in silico* prediction to experimental verification and early-phase clinical testing (Reza et al., 2019; Li et al., 2022; Ojha et al., 2025). In addition to compound discovery, AI-based approaches are increasingly being used to tackle one of the most pressing challenges in antibiotic development: resistance and drug escape. Additionally, evolutionary modeling tools combine the genomic diversity of bacteria, mutation rates, selective pressures, and fitness costs to predict how pathogens could develop resistance to new antibiotics. ML-based simulations of the fitness landscape have made successful predictions of resistance-conferring mutations identified in experimental evolution studies, showing that AI can predict evolutionary outcomes rather than react to them (Petrungaro et al., 2021; Feng et al., 2022; Babirye et al., 2024; Sufi, 2025). This predictive capability enables the design of drug targets with reduced evolutionary escape potential and the development of new compounds that have higher fitness costs for resistant strains, thus extending the therapeutic lifetime. However, major challenges persist in the complete utilization of ML and AI in antibiotic discovery. Data bias is a major concern, as existing antimicrobial datasets are often small, imbalanced, and biased toward well-studied chemical structures or bacterial species, thus increasing the risk of overfitting and inflated performance metrics. In addition, generalization to various pathogens, resistance mechanisms, and chemical structures is still challenging, especially when trained on *in vitro* activity data that do not necessarily generalize to *in vivo* activity (Bilal et al., 2025; Alharbi, 2025). A critical requirement for improving translational robustness in AI-driven antibiotic discovery is the implementation of out-of-distribution (OOD) generalization testing as a mandatory component of the computational workflow rather than an optional validation step (Wu et al., 2025). Many antimicrobial prediction models demonstrate excellent internal performance on benchmark datasets but fail substantially when applied to unseen bacterial species, novel resistance mechanisms, or chemically distinct scaffolds outside the training distribution (Stokes et al., 2020). This problem is particularly evident in models trained predominantly on well-characterized pathogens, such as *Escherichia coli*, *S. aureus*, or *Pseudomonas aeruginosa*, where predictive performance often declines sharply for emerging or underrepresented pathogens (Xu et al., 2026). Representative case studies, such as the halicin discovery pipeline demonstrated stronger translational relevance because predictions were validated against structurally novel compounds and independent biological assays rather than relying solely on conventional training-test splits (Stokes et al., 2020). Similarly, β-lactamase inhibitor prediction studies using cross-species validation datasets have shown that external validation across diverse enzyme families provides a more realistic estimate of clinical applicability than random split benchmarking. Therefore, robust OOD validation using independent datasets, phylogenetically distant pathogens, resistance-evolution simulations, and prospective wet-lab testing should be treated as a standard requirement in computational antibiotic discovery pipelines to ensure that predictive models are genuinely generalizable, biologically meaningful, and clinically translatable (Pitakbut et al., 2024; Ghouch et al., 2025). Furthermore, many of the most successful deep learning models are inherently “black box” predictors, which can undermine confidence in the results among experimental biologists and clinicians. Recent advances in healthcare AI highlight the shift from static prediction models to agentic AI systems capable of reasoning, planning, and tool-assisted decision-making. Agentic large language models (LLMs) integrate external databases, clinical guidelines, and decision-support tools to perform multi-step problem solving rather than single-output prediction. In antimicrobial discovery, such frameworks can combine resistome analysis, structural modeling, ADMET prediction, resistance evolution simulation, and experimental feedback within a unified pipeline. This improves target prioritization, reduces prediction errors, and enhances explainability through traceable stepwise reasoning, making agentic AI a promising future direction for clinically translatable antibiotic discovery (Yuan, 2025).

**FIGURE 5:**
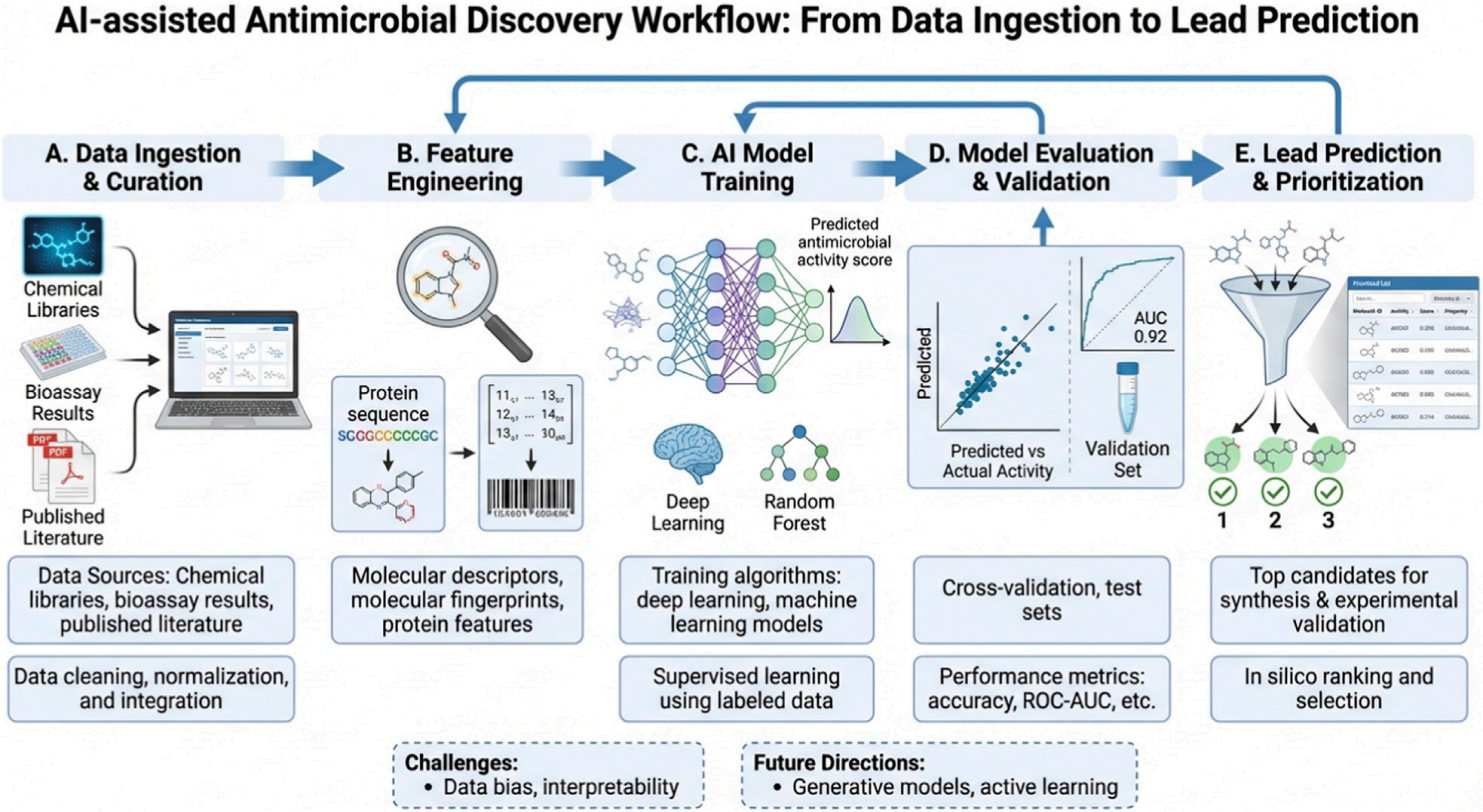
Overview of an AI-assisted antimicrobial discovery workflow from data ingestion to lead prediction. The process begins with integrating chemical libraries, bioassay results, and published literature, followed by data cleaning and normalization. Feature engineering generates molecular descriptors, fingerprints, physicochemical properties, and protein sequence features. These data train AI/ML models, including deep learning and random forest algorithms, to predict antimicrobial activity. Model performance is validated using cross-validation and ROC-AUC metrics. Top-ranked candidate compounds are prioritized for synthesis and experimental testing, accelerating identification of promising antimicrobial lead molecules efficiently.

To overcome this challenge, there is currently active research in the development of “explainable AI” methods, such as attention models, feature attribution techniques, and concept-based explanation techniques, which aim to elucidate the molecular substructures, physicochemical properties, or genomic elements that underlie model predictions (Gangwal and Lavecchia, 2026). Improving the interpretability of these approaches will not only increase confidence and adoption but also enable hypothesis generation, target validation, and rational compound optimization. Taken together, the increasing number of experimentally validated case studies demonstrates that ML and AI have already transitioned from theory to practice and have provided real-world advances in antibiotic discovery, including the development of new antibacterial agents, resistance modulators, and evolution-informed design strategies (Stokes et al., 2020; Cesaro et al., 2023a; Lin et al., 2025; Wu et al., 2025). When combined with genomic information, systems biology, evolutionary modeling, and experimental validation, ML- and AI-based strategies provide a paradigm-shifting platform for accelerating antibiotic discovery and combating the global crisis of AMR (Figure 5) (Table 3).

**TABLE 3:** Critical limitations of AI-driven antibiotic discovery models: challenges, translational barriers, and the role of explainable artificial intelligence (XAI) (Moffat et al., 2017; Stokes et al., 2020; Walters and Murcko, 2020; Gangwal and Lavecchia, 2026).

AI/ML challenge	Data bias	Lack of generalizability	Black-box prediction problem	Limited experimental validation	Poor quality negative data	Chemical space limitation	Overfitting	Poor integration with ADMET	Resistance evolution not modelled	Lack of explainable AI adoption
Core Problem	Training datasets are skewed toward known compounds and well-studied pathogens	Models perform well on training species but fail on unseen pathogens	Deep learning models provide predictions without mechanistic explanation	Many AI predictions remain computational only	Inactive compounds are poorly represented	AI models explore known chemistry rather than novel scaffolds	Model learns noise rather than biological principles	Activity prediction without pharmacokinetic relevance	Models predict activity but not future resistance escape	XAI tools are underused in antimicrobial discovery
Underlying Cause	Overrepresentation of common scaffolds (e.g., β-lactams), limited negative datasets, publication bias	Species-specific training and narrow assay conditions	Hidden-layer complexity and poor interpretability	Lack of funding, wet-lab resources, or reproducible workflows	Publication bias toward positive results	Dependence on historical training libraries	Small datasets and excessive model complexity	Separate modeling of potency and safety	Lack of evolutionary fitness modeling	Focus on prediction accuracy over interpretability
Impact on Antibiotic Discovery	Models preferentially predict known chemistry rather than novel antibiotics	Poor transferability across Gram-positive, Gram-negative, and MDR pathogens	Difficult to understand why a compound is predicted active	Large gap between prediction and therapeutic application	Weak discrimination between active and inactive molecules	Reduced discovery of structurally distinct antibiotics	Excellent internal performance but poor real-world results	Potent compounds fail due to toxicity or permeability issues	Short-lived antibiotic efficacy	Limits trust from clinicians and experimental biologists
Example in Antibiotic Research	Halicin discovery highlighted rarity of identifying structurally novel compounds	Model trained on *E. coli* often underperforms for *A. baumannii* or *M. tuberculosis*	AI predicted halicin before mechanism was known experimentally	Many docking-based β-lactamase inhibitors fail during biological testing	Many models classify inactive compounds incorrectly	Traditional docking often misses non-classical molecules like halicin	High ROC-AUC but failed external validation	Many predicted Gram-negative hits fail due to permeability barriers	Initial potent leads rapidly lose efficacy clinically	Strong predictions remain unused without mechanistic rationale
Consequence for Translational Adoption	Limits discovery of first-in-class antibiotics	Limits broad clinical utility	Clinicians and microbiologists hesitate to trust predictions	Reduces trust in computational pipelines	Inflates performance metrics artificially	Limits innovation against resistant pathogens	Loss of translational trust	Delays translation to lead optimization	Weak long-term clinical value	Slows translational adoption significantly
Effect on Clinical Reliability	Reduced confidence in predictions for new pathogens	Poor reliability in real-world infections	Lower clinical adoption	Low translational credibility	Unstable clinical prediction	Reduced novelty for clinical pipelines	Unstable clinical predictions	Unsafe or clinically unusable candidates	Unstable therapeutic durability	Reduced clinician confidence
Risk of False Positives/Negatives	High false positives for familiar scaffolds; false negatives for novel chemotypes	High false negatives in new bacterial species	Unknown prediction errors remain hidden	Very high false discovery rate	High false positives	False negatives for unconventional molecules	Both false positives and false negatives increase	False positives in activity-only models	False optimism in candidate selection	Hidden biases remain unresolved
Influence on Regulatory Approval	Regulatory concerns over model robustness	Weakens confidence for approval of broad-spectrum leads	Regulatory agencies require mechanistic confidence	Strong barrier to regulatory acceptance	Weakens reproducibility and validation confidence	Novelty is essential for patentability and approval	Poor regulatory reproducibility	Safety concerns delay approval	Resistance management is central to approval	Major regulatory limitation
Effect on Experimental Validation	Wet-lab validation often fails for “predicted actives” lacking true novelty	Requires repeated re-training for each pathogen	Experimental design becomes difficult without rationale	High attrition in preclinical testing	Misleads medicinal chemistry optimization	Repeated rediscovery of old scaffolds	Failed independent validation studies	Late-stage failure in animal models	Resistance emerges after deployment	Harder prioritization of candidates
Representative Algorithms Affected	RF, SVM, DNN, CNN	DNN, Graph Neural Networks, Transformer models	Deep Neural Networks, GANs, GNNs	All ML pipelines	RF, SVM, QSAR, DNN	QSAR, classical ML, docking-assisted AI	DNN, CNN, GNN	Docking + ML pipelines	Classical QSAR, docking-first pipelines	Especially DNN, GANs, GNNs
Possible Mitigation Strategy	Balanced datasets, external validation, negative sampling	Diverse cross-species datasets and federated learning	Explainable AI, attention maps, SHAP, lime	Mandatory integrated *in vitro* + *in vivo* validation	Curated inactive datasets and failed trial integration	Generative AI, *de novo* design, reinforcement learning	Cross-validation, independent external datasets	Multi-objective optimization including ADMET	Evolution-aware ML and fitness landscape prediction	Routine integration of SHAP, lime, attention models
Role of Explainable AI (XAI)	XAI identifies whether predictions rely on true biological features or dataset bias	XAI helps identify whether prediction is target-driven or species-specific artifact	Central role-reveals molecular substructures and target rationale	XAI prioritizes strongest mechanistic candidates for testing	XAI helps identify overfitting to positive-only datasets	XAI helps evaluate whether novelty retains biological plausibility	XAI reveals unstable features driving predictions	XAI clarifies trade-offs between potency and toxicity	XAI can identify mutation-prone binding regions	Essential for bridging AI prediction to translational biology
Recommended Validation Approach	Independent multi-pathogen validation	Independent pathogen-specific validation	Mechanism-of-action validation using biochemical assays	MIC, enzyme inhibition, infection models	Blind validation with prospective testing	Orthogonal validation across chemical classes	Multi-center independent testing	Parallel efficacy + toxicity validation	Serial passage and resistance evolution studies	Mechanism-supported experimental validation
Long-Term Research Need	Creation of larger unbiased antimicrobial databases	Pan-pathogen benchmarking frameworks	Development of interpretable deep learning frameworks	Standardized benchmark-to-validation pipelines	Public databases of failed antibacterial candidates	Expansion of generative chemistry platforms	Standardized benchmarking standards	Unified AI models combining activity + ADMET	Predictive resistance modeling frameworks	Establishing XAI as a standard in antibiotic AI pipelines

## 7 ADMET-driven lead optimization for resistance-resilient antibiotics

*In silico* prediction of ADMET properties has thus become an integral part of antibiotic lead optimization, especially in AMR, where properties of compounds, bacterial barriers, and host safety considerations all play a role in determining success. In addition, *in silico* ADMET screening in the early stages of lead optimization allows for the selection of compounds with favourable ADMET properties before embarking on costly experimental work (Absar et al., 2025; Zhang J. et al., 2025). Computational models of intestinal absorption and membrane permeability, based on molecular properties, such as lipophilicity, polar surface area, and hydrogen bonding ability, have been used to ensure sufficient systemic availability and penetration into infected tissues, as well as addressing the permeability problem posed by the outer membranes of Gram-negative bacteria (Subramanian and Douglas, 2006; Dahlgren and Lennernäs, 2019). For example, the optimization of new β-lactamase inhibitors discovered by virtual screening has relied on *in silico* models of permeability and porin uptake to balance molecular size and polarity, leading to improved intracellular uptake and restored antibacterial efficacy against resistant strains (Ehmann et al., 2012; Richter et al., 2017). Further, toxicity prediction is one of the most important applications of *in silico* ADMET tools. ML classifiers trained on data sets related to hepatotoxicity, cardiotoxicity, and genotoxicity help in the early screening of compounds with potential adverse effects, thus improving safety margins. Case studies related to antibiotic repositioning demonstrates the utility, where computational toxicity filtering successfully eliminated candidates with predicted hERG inhibition or mitochondrial toxicity before *in vivo* evaluation, thus shortening the timeline for the experimental validation of safer repositioned antibiotics (Mayr et al., 2016; Pushpakom et al., 2019; Boysen et al., 2021; Mostafa et al., 2024). Additionally, metabolic stability prediction is another lead optimization tool that helps in simulating host-target interactions, specifically with host cytochrome P450 enzymes, and predicting clearance rates, half-life, and metabolite production. *In silico* metabolism modeling has been a key tool in optimizing fragment-based antibacterial leads by simulating structural modifications to overcome rapid metabolic instability while retaining antibacterial efficacy (Tyzack and Johannes, 2019; Zhai et al., 2023). In addition to traditional host-focused ADMET concerns, optimization against bacterial resistance mechanisms has become a hallmark of modern antibacterial design. Computational models predicting susceptibility to efflux pumps have allowed rational design modifications to overcome active efflux; as illustrated in case studies, virtual screening-derived efflux liability scores guided chemical optimization, resulting in improved intracellular persistence and restored antibacterial efficacy against multidrug-resistant bacteria (McDowell et al., 2019; Akshay et al., 2023; Sierra-Hernández et al., 2026). Concurrently, the ability to predict the stability of enzymes *in silico* has been utilized to design antibiotics and resistance inhibitors that are less vulnerable to degradation by bacterial enzymes, such as β-lactamases and modifying enzymes, by identifying susceptible chemical structures and replacing them with more stable ones (Davies and Martin, 2021; Pratibha et al., 2026). A study highlights major resistance mechanisms including β-lactamases, efflux pumps, target modification, and biofilm formation, while emphasizing computational resources such as molecular docking, structure-based drug design, and *in silico* screening for identifying novel antimicrobial targets and overcoming MDR (Elshobary et al., 2025). Further, biofilm penetration is another challenge that is of paramount importance in the clinic, since biofilm-related infections are notoriously resistant to antibiotics because of limited diffusion and changes in bacterial physiology. Computer models that consider molecular size, charge, and hydrophobicity have been used to predict biofilm penetration ability, and examples show that virtual optimization of these properties can lead to compounds with improved activity against bacteria in biofilms, which have been validated in subsequent experimental biofilm studies (An et al., 2021; Zafer et al., 2024). These examples demonstrate that ADMET prediction *in silico* is not limited to host pharmacokinetics and toxicity but also includes bacterial-specific barriers and resistance mechanisms. When combined with virtual screening, molecular docking, machine learning-based activity prediction, and evolutionary modeling, ADMET-informed lead optimization enables a systems-level approach to antibiotic discovery (Serrano et al., 2024). Notably, empirical case studies have shown that molecules optimized using ADMET simulations are more likely to possess synergy in efficacy, safety, and resistance resilience, thus supporting the importance of ADMET simulations as a key interface between target identification and the development of effective antimicrobials in combating antimicrobial resistance (Table 4).

**TABLE 4:** Representative case studies of *in silico*-driven antibiotic discovery pipeline**s**.

Study	Target/Strategy	*In Silico* approach	Tool/Model	Software/Platform	Experimental outcome	Key contribution
Stokes et al. (2020)	Broad-spectrum antibacterial	Deep learning screening	Neural network	TensorFlow	Halicin active vs. MDR bacteria	AI-first antibiotic discovery
	Narrow-spectrum antibiotics	ML-guided screening	Deep learning	TensorFlow	Species-selective antibiotics	Precision antimicrobial design
	Antibacterial QSAR	Ligand-based screening	Random forest	MOE	Novel antibacterial leads	Early ML in antibiotics
	Efflux pump inhibitors	Pharmacophore + QSAR	SVM	Schrödinger	Restored antibiotic sensitivity	Resistance modulation
	NDM-1 β-lactamase	Structure-based docking	Docking + MD	AutoDock, GROMACS	β-lactamase inhibition	Resistance enzyme targeting
	Metallo-β-lactamase	SBVS	Glide XP	Schrödinger	Synergistic β-lactam rescue	Structure-guided inhibition
	Novel antibacterials	Generative AI	GAN	DeepChem	*De novo* active compounds	AI molecular generation
	Antibacterial scaffolds	Fragment-based design	Docking + FBDD	GOLD	Fragment-derived inhibitors	Fragment efficiency
	Allosteric enzyme sites	MD-driven screening	Enhanced MD	AMBER	Allosteric inhibitors	Dynamic pocket discovery
	Antibacterial peptides	ML prediction	Deep learning	Keras	Potent AMPs	Non-classical antibiotics
	Gram-negative penetration	ML ADMET	Gradient boosting	scikit-learn	Improved permeability	ADMET-driven optimization
	*De novo* antibiotics	SMILES generation	RNN	PyTorch	Novel chemical space	Generative chemistry
Liu et al. (2021)	Biofilm inhibitors	Network pharmacology	Graph analysis	Cytoscape	Biofilm disruption	Systems-level targeting
	Resistance enzymes	Docking + rescoring	Consensus scoring	AutoDock Vina	Reduced false positives	Improved SBVS reliability
	Antibiotic optimization	Bayesian optimization	BO	Python	Faster lead optimization	Closed-loop design
	Repurposed antibiotics	AI drug repurposing	ML classifier	DeepChem	Rapid hit identification	Accelerated translation
	MDR tuberculosis	ML screening	CNN	TensorFlow	New anti-TB hits	Pathogen-specific AI
	Efflux evasion	Physchem ML models	Random forest	RDKit	Increased intracellular drug levels	Resistance-aware design
	Enzyme stability	MD + docking	Free energy calc	GROMACS	Improved enzymatic stability	Resistance resilience
	Resistance evolution	Evolution-aware ML	Fitness landscape	Custom ML	Predicted escape mutations	Anticipatory resistance modeling
	Combination therapy	Systems pharmacology	Network ML	MATLAB	Synergistic drug pairs	Combination optimization
	Host–pathogen targets	Multi-omics AI	Graph neural network	PyTorch Geometric	Dual-target antibiotics	Integrated systems discovery

## 8 From *in silico* predictions to translational validation in antibiotic discovery

The integration of computer-assisted drug discovery with *in-vitro* studies and subsequent clinical use is an important critical juncture in antibiotic discovery. Computer predictions need to be validated and optimized by rigorous wet lab experiments and translation. The typical pipeline involves initial *in vitro* validation, including compounds identified by computer screening, ML algorithms, or ADMET filtering are tested for antibacterial properties, ascertained by minimum inhibitory concentrations, and defined by mechanisms, namely enzyme inhibition, efflux, or biofilm disruption (Abdelmalek et al., 2024; Oselusi et al., 2024; Sanapalli et al., 2026b). There are numerous examples of the efficacy of this pipeline. For instance, AI-identified antibacterial compounds have progressed from computer screening to *in vitro* growth inhibition assays and have shown potent activity against multidrug-resistant bacteria. Likewise, SBVS for β-lactamases or other essential metabolic enzymes has identified compounds whose binding affinity was validated by biochemical assays and bacterial susceptibility testing, optimizing the docking and scoring pipeline (Pájaro-Castro et al., 2026). Once validated *in vitro*, the lead compounds are taken to animal infection models. These experiments provide vital information on efficacy, toxicity, and pharmacokinetics in a living organism, allowing an estimate of how the compounds would work in a biological system (Wang L. et al., 2024). Moreover, *in silico*-discovered antibiotics and resistance modulators have shown therapeutic efficacy in mouse models of systemic and localized infections, with decreases in bacterial loads and survival rates consistent with computational predictions and *in vitro* activity. These results underscore the importance of animal models in determining whether desirable *in silico* properties are reflected in tangible therapeutic benefit in the context of complex host-pathogen interactions (Stokes et al., 2020; El-Sayed et al., 2026). However, significant translational hurdles remain, making the successful translation of computationally discovered candidates into therapeutic agents challenging. One of these hurdles is pharmacokinetic-pharmacodynamic (PK/PD) discordance, where agents with high *in vitro* antibacterial potency lack sufficient concentrations at the infection site or are rapidly cleared from the body, thus reducing their therapeutic potential (Zampieri et al., 2018). Results from early-case evaluations of antibiotic candidates suggest that poor tissue penetration, unfavourable distribution, and rapid metabolism can compromise promising candidates, emphasizing the importance of early integration of PK/PD modeling and optimization based on both computational and experimental approaches (Bissantz et al., 2024; Talha and Roque-Borda, 2026). Regulatory hurdles also make translation difficult, since antibiotics must meet rigorous safety, efficacy, and resistance management standards, and new mechanisms of action or atypical chemical structures may face additional scrutiny in the regulatory process. Further, repurposed drugs may utilize prior safety data; however, their respective dosage, formulation, or intellectual property may pose challenges (Muteeb et al., 2023; Halawa et al., 2024). In contrast, *de novo* AI-designed compounds will necessitate extensive toxicological and clinical evaluations. Most importantly, the successful translation of drug candidates from investigator-led research to clinical practice increasingly relies on integrated workflows where *in silico* predictions are used to guide the design of experiments, and experimental results are used to refine the computational model, and both are used to inform regulatory strategies throughout each phase of the development process (Serrano et al., 2024; Goryanin et al., 2025; Pathak et al., 2025; Sanapalli et al., 2026b). The case studies described herein demonstrate the iterative feedback loop associated with computationally predicted outcomes being continuously updated based on the findings from *in vitro* and *in vivo* studies provide an example and a possible pathway to overcoming key barriers to translation. Collectively these examples demonstrates that although *in silico* methods provide a significant speed-up in antibiotic discovery processes, the ultimate impact of these technology platforms is dependent on whether the experimental validation results match the computationally predicted results and on the ability of the research to rigorously assess PK/PD parameters of the antibiotic candidate and actively engage with regulators to ensure that the candidates identified through computation can be developed into effective clinical therapies for the treatment of infections caused by antibiotic-resistant bacteria (Table 5).

**TABLE 5:** Translational pipeline from *In Silico* antibiotic discovery to clinical validation.

Stage of pipeline	Primary objective	Key computational/Experimental methods	Data generated	Validation readouts	Representative outcomes/Case evidence	Major challenges/Bottlenecks	Translational significance	References
Target identification (*in silico*)	Identify essential bacterial or resistance-associated targets	Genome-scale metabolic modeling, essentiality analysis, protein–protein interaction networks, resistance gene databases	Target prioritization lists, essentiality scores	Conservation, druggability, resistance relevance	Identification of β-lactamases, efflux pumps, and essential metabolic enzymes	Target redundancy, compensatory pathways	Determines biological relevance and feasibility	King et al. (2014); Jumper et al. (2021)
Structure acquisition	Enable structure-based discovery	X-ray crystallography, cryo-EM, homology modeling, AlphaFold predictions	3D protein structures	Resolution, structural confidence	High-quality structures of NDM-1, VIM, PBPs	Structural flexibility, missing loops	Enables SBVS and rational design	Baek et al. (2021); Jumper et al. (2021)
Virtual screening	Identify candidate antibacterial compounds	SBVS, LBVS, AI/ML models, ADMET filtering	Docking scores, predicted affinities	Binding pose stability, ranking consistency	AI-identified antibacterial hits active vs. MDR pathogens	False positives, scoring inaccuracies	Rapid hit identification	Ferreira et al. (2015)
Hit prioritization	Reduce chemical space to testable candidates	Consensus scoring, ML re-ranking, physicochemical filtering	Shortlisted compounds	Chemical novelty, predicted selectivity	Optimization of docking pipelines for β-lactamase inhibitors	Overfitting, bias in models	Improves experimental success rate	Rastelli and Pinzi (2019); Cesaro et al. (2023b)
*In vitro* antibacterial screening	Validate predicted antibacterial activity	MIC determination, time-kill assays, growth inhibition curves	MIC values, bactericidal vs. bacteriostatic profiles	Potency against susceptible and MDR strains	AI-predicted compounds showing low MICs	*In vitro*-*in vivo* disconnect	First biological validation	Jendrzejewska and Karwowska, (2022)
Mechanism-of-action studies	Confirm predicted mode of action	Enzyme inhibition assays, efflux assays, biofilm assays	IC_50_, efflux inhibition rates	Target engagement confirmation	Docking-predicted β-lactamase inhibitors validated biochemically	Off-target effects	Confirms computational predictions	King et al. (2014)
Resistance profiling	Assess resistance emergence risk	Serial passage, mutation frequency assays	Resistance rates	Genetic stability	Identification of low-resistance-potential candidates	Rapid adaptive resistance	Supports durability assessment	Feng et al. (2022)
Lead optimization (iterative)	Improve potency and selectivity	Docking refinement, MD simulations, SAR modeling	Optimized analogs	ΔG improvements, stability metrics	Iterative refinement guided by *in vitro* data	Optimization trade-offs	Core feedback loop stage	Neto et al. (2020)
ADMET prediction (*in silico*)	Pre-empt toxicity and PK issues	ML-based ADMET models, PBPK simulations	PK/PD predictions	Absorption, clearance estimates	Early elimination of poor PK candidates	Model inaccuracies	Reduces late-stage attrition	Daina et al. (2017)
*In vivo* efficacy studies	Validate therapeutic benefit	Mouse infection models (systemic/local)	Bacterial load reduction, survival	CFU reduction, survival curves	AI-discovered antibiotics effective in murine models	Species differences	Confirms translational relevance	King et al. (2014)
*In vivo* toxicity	Assess safety profile	Acute and sub-chronic toxicity studies	LD_50_, organ toxicity	Therapeutic window	Identification of tolerable dose ranges	Hidden toxicities	Essential for clinical feasibility	Stokes et al. (2020)
PK/PD evaluation	Link exposure to efficacy	Plasma/tissue PK studies, PK/PD modeling	Cmax, AUC, T>MIC	PK/PD target attainment	Identification of PK/PD discordance	Poor tissue penetration	Major translational hurdle	Mouton et al. (2012)
PK/PD optimization	Improve *in vivo* performance	Structural modification, formulation changes	Optimized exposure profiles	Improved efficacy at lower doses	Salvaging potent but unstable leads	Formulation complexity	Bridges lab-to-clinic gap	Nielsen and Friberg, (2013)
Regulatory preclinical alignment	Prepare for IND-enabling studies	GLP toxicology, resistance risk assessment	Regulatory datasets	Compliance with guidelines	Early regulator engagement	Stringent regulatory scrutiny	De-risks clinical transition	Theuretzbacher et al. (2020)
Repurposed drug translation	Accelerate development	Leveraging existing safety data	Clinical repositioning data	Dose feasibility	Rapid entry into trials	IP and dosing limitations	Shortens timelines	Aloni-Grinstein et al. (2025)
*De novo* AI-designed translation	Advance novel scaffolds	Full toxicology and safety packages	IND-ready data	Safety margins	Halicin-like trajectories	High development cost	Enables first-in-class agents	Zhan et al. (2025)
Iterative feedback integration	Improve prediction accuracy	Model retraining with experimental data	Updated algorithms	Prediction-outcome concordance	Continuous refinement loops	Data integration challenges	Hallmark of modern pipelines	Cesaro et al. (2023b)
Clinical trial readiness	Enable human testing	Phase 0/I trial design	Clinical protocols	Safety and PK endpoints	Translation of validated candidates	Trial cost, recruitment	Determines real-world impact	Piddock et al. (2024b)

### 8.1 Clinical implementation challenges and real-world translation barriers

Despite major advances in *in silico* antibiotic discovery, successful translation into routine clinical practice remains challenging. Computationally predicted compounds that demonstrate promising *in vitro* and *in vivo* activity frequently encounter substantial barriers during clinical development, regulatory approval, and real-world implementation (Xu et al., 2026). These challenges arise from the complexity of infectious diseases, pathogen heterogeneity, host-pathogen interactions, and the stringent safety requirements associated with antimicrobial therapy. One of the major barriers is pharmacokinetic and pharmacodynamic (PK/PD) inconsistency between computational predictions and human clinical performance (Payne et al., 2007). Compounds with excellent docking scores and strong antibacterial activity may fail due to inadequate tissue penetration, poor bioavailability, rapid metabolic clearance, limited intracellular accumulation, or inability to reach therapeutic concentrations at infection sites. This is particularly relevant for infections involving biofilms, intracellular pathogens, or poorly vascularized tissues, where effective drug delivery becomes difficult despite favourable *in silico* predictions (Kim T. et al., 2025; Garcia et al., 2026). Another major challenge involves bacterial evolution and the rapid emergence of resistance during clinical use. Even compounds designed against conserved essential targets may become vulnerable to resistance through compensatory mutations, horizontal gene transfer, efflux pump upregulation, or adaptive metabolic rewiring (Jiang et al., 2019; Windels et al., 2019). Predicting long-term evolutionary escape remains difficult, and many promising candidates fail because resistance emerges faster than anticipated during clinical deployment. Further, regulatory approval also presents significant obstacles. Antibiotics require rigorous demonstration of safety, efficacy, microbiological relevance, and resistance management strategies. Novel compounds generated through AI-based design or *de novo* molecular generation often possess unconventional chemical scaffolds, which may face additional regulatory scrutiny due to limited historical safety data (Xu et al., 2026). Similarly, repurposed drugs may require new dosage optimization, formulation changes, and additional clinical trials despite prior approval for non-infectious indications. Additionally, economic and commercial barriers further limit clinical implementation. Antibiotic development offers lower financial returns compared with chronic disease therapeutics, resulting in reduced pharmaceutical investment and frequent withdrawal of industry support (Stokes et al., 2020). Even scientifically promising candidates may fail to progress due to limited market incentives and stewardship policies that intentionally restrict antibiotic use. Finally, successful implementation requires integration of computational predictions with clinical microbiology, rapid diagnostics, antimicrobial stewardship programs, and personalized treatment strategies (Cesaro et al., 2025). Future progress will depend on multidisciplinary collaboration among computational scientists, microbiologists, clinicians, pharmacologists, and regulatory agencies to ensure that *in silico* discoveries are translated into robust, clinically relevant, and resistance-resilient antimicrobial therapies.

## 9 Opportunities, pitfalls, and ethics of AI-driven antibiotic discovery

Although there are rapid development and increased impact from *in silico* antibiotic discovery pipelines that target antibiotic resistance, there are still several scientific, technical, and ethical barriers that continue to hinder their overall robustness, generalizability, and capable deployment (Giacobbe et al., 2026). A major limiting factor is the quality of data used for training computational models, as they are inherently dependent on dataset quality for training and validation. For example, the antimicrobial dataset is often incomplete, biased to well-studied pathogens or chemical scaffolds, and is compiled from heterogeneous experimental conditions. Variations in assay designs, media, ​bacterial strain, and endpoints can introduce noise and systematic bias into the dataset, causing artificially inflated predictive performance, which fails to accurately translate to different laboratories or biological contexts (Kasse et al., 2025; Mehran et al., 2026). A lack of adequate negative data (i.e. no resistant bacterial strain), inconsistent annotation of resistance mechanisms, and absence of emerging pathogens also play a major role in reproducibility issues. Therefore, rigorous curation of datasets, standardized benchmarking of models, transparent reporting of model performance, and independent external validation of models are necessary to support scientifically sound and translationally meaningful *in silico* predictions (Imchen et al., 2020; Yamin et al., 2023). The second major barrier is transparency and interpretability of AI used in ML and deep learning models for antibiotic discovery; therefore, most of the high-performing models being used in antibiotic discovery based on DNN and generative architecture function as “black boxes” (can provide accurate predictions without providing clear mechanistic explanations). Further, insufficient transparency surrounding biological interpretation creates barriers to generating hypotheses, which, in turn, leads to decreased confidence between practitioners (e.g., researchers, clinicians and regulatory bodies) (Ding et al., 2025; Qadri et al., 2025). For antibiotic discovery, understanding both the mechanisms by which drugs act, as well as the ways in which bacteria evolve to resist drugs, is paramount. In addition, opaque predictive models may impede rational optimization and lead to unrecognized off-target effects, unintended selective pressures on the target population, and compromised investigational effort. The emergence of the field of explainable AI is aimed at overcoming these limitations by providing researchers with insight into the molecular characteristics, genomic signals (e.g., sequence, gene expression), and structural motifs that inform their predictions (Proietti et al., 2024; Jarallah et al., 2025; Qadri et al., 2025). Despite many interpretive models being qualitative or context-specific in nature, a clear need exists for the establishment of standardized (preferably biological) explainability frameworks for the application of these technologies to antimicrobial research (Moon et al., 2024). Furthermore, dual use presents additional ethical challenges aside from the limitations associated with technological capability. In particular, computational tools which are able to rapidly design and/or optimize bioactive molecules can potentially be misused and facilitate the creation of malicious biological agents or increase the virulence or resistance potential of pre-existing pathogens; therefore, the same algorithmic process being applied for the design and discovery of antimicrobial agents could theoretically also be used for the design of molecules that will be able to evade treatment or accelerate the development of microbial resistance (Urbina et al., 2022; Lima et al., 2024; Wheeler, 2025; Pannu et al., 2025). There should be a lot of attention paid to this dual-use potential to ensure it is managed correctly, that data is shared responsibly, and that biosecurity policies are consistent with current biosecurity policies. As well, any ethics oversight that is provided should include a risk assessment of computer-generated outcomes, controlled access to sensitive data, and engagement with policymakers to prevent misuse of bioinformatics/AI advances (Cummings et al., 2021; Sun et al., 2022). The last implicit ethical area associated with *in silico* antibiotic discovery is the issue of equitable access and global relevance. Models developed primarily on data collected from high-income countries may not function as well for strains of pathogens, resistance patterns or clinical situations that prevail in low and middle-income countries, where the bulk of the burden of AMR is present. Addressing this disparity will require inclusive data generation, international collaboration, and open-science practices that have a priority to positively impacting global health (Cesaro et al., 2025; Mulat et al., 2025; Salama et al., 2026). In conclusion, while *in silico* pipelines represent unprecedented opportunities to accelerate the discovery of antibiotics, their commercialization will only happen if the quality/reproducibility of the input data is analysed; the transparency of AI is improved; and the ethical and dual-use potential risks are proactively managed to ensure ethical practices are reflected in the development and use of computational technologies. The successful commercialisation of these technologies will require a combination of the technical rigor associated with traditional science and the ethical ability to make their use and implementation safe, effective and provide a globally beneficial solution to combatting AMR.

## 10 Critical appraisal, methodological constraints, and translational limitations of *in silico* antibiotic discovery reviews

Although this review provides a comprehensive overview of contemporary *in silico* antibiotic discovery pipelines targeting AMR, several limitations should be acknowledged. First, the review follows a structured narrative approach rather than a formal systematic review or meta-analysis. Although major biomedical and computational databases were searched using predefined keywords and inclusion criteria, the selection and interpretation of studies may still be influenced by publication bias and the availability of high-impact or well-cited reports (Sterne et al., 2008; Snyder, 2019). As a result, some relevant emerging studies, particularly unpublished data or recent preprints, may not have been included. Second, the rapidly evolving nature of computational drug discovery, particularly in AI, ML, and protein structure prediction, means that new tools, algorithms, and validation studies continue to emerge at a pace that may quickly outdate specific examples discussed in this review (Bender and Cortés-Ciriano, 2021). Technologies, such as generative AI, large language models for molecular design, and next-generation structure prediction platforms are advancing rapidly and may further reshape the field beyond the current scope of this manuscript. Third, while this review emphasizes experimentally validated case studies, such as halicin discovery, β-lactamase inhibitor development, and efflux pump inhibitor screening, many published *in silico* predictions remain insufficiently validated *in vivo* or in clinical settings (Bae et al., 2025). Therefore, the translational potential of several computational strategies discussed here should be interpreted cautiously, as strong docking scores or favourable ADMET predictions do not always translate into clinical efficacy (Zhou and Tao, 2026). Finally, this review primarily focuses on bacterial antibiotic resistance and excludes detailed discussion of antifungal, antiviral, and antiparasitic resistance mechanisms, which may also benefit from similar computational discovery frameworks (Fisher et al., 2022). In addition, economic, regulatory, and global accessibility challenges were discussed conceptually but not quantitatively analyzed. Despite these limitations, the review provides an integrated and up-to-date framework for understanding how genome-informed and data-driven computational strategies can support the future development of resistance-resilient antimicrobial agents (Bender and Cortés-Ciriano, 2021).

## 11 Future perspectives

The rapidly accelerating convergence of bioinformatics, AI, and systems biology is ushering in a new era of digital-first antibiotic discovery, where computational methods are becoming the primary drivers of hypothesis generation, target identification, and lead optimization for antimicrobial development (Wong et al., 2024). Unlike traditional antibiotic discovery approaches that rely heavily on empirical screening and trial-and-error experimentation, digital-first strategies integrate genomics, structural biology, ML, and predictive modeling to systematically explore vast chemical and biological spaces. This transition is particularly important because the pace of AMR evolution continues to outstrip the rate at which new antibiotics are developed using conventional methods (Payne et al., 2007). Further, the expansion of high-throughput sequencing and real-time pathogen genomic surveillance is enabling dynamic mapping of bacterial genomes, resistomes, and pan-genomes, allowing computational models to rapidly identify emerging resistance mechanisms and prioritize targets with lower evolutionary escape potential (Hu et al., 2024). This creates opportunities for designing resistance-resilient therapeutics and combination strategies before resistance becomes clinically widespread. Future antibiotic discovery platforms are expected to integrate genomics, AI, and systems biology into unified and interoperable computational ecosystems capable of generating actionable predictions for target vulnerability, compound activity, and resistance evolution (Stokes et al., 2020). Furthermore, multi-omics integration, including transcriptomics, proteomics, metabolomics, and metagenomics, will provide deeper insights into host-pathogen interactions, metabolic adaptation, bacterial stress-response pathways, and hidden vulnerabilities within microbial networks. This systems-level understanding will move antibiotic development beyond the traditional single-target paradigm toward network-based therapeutic strategies that account for biological redundancy, compensatory pathways, and adaptive resistance mechanisms (Hendriksen et al., 2019). Such approaches will improve the identification of synergistic drug combinations and non-obvious therapeutic targets. Additionally, an equally important future direction is the development of personalized antimicrobial therapy. By combining rapid sequencing technologies with AI-based clinical decision-support systems, clinicians may soon be able to select antibiotics based on the molecular characteristics of the infecting pathogen, its resistance signature, and the host’s genomic and immunological profile (Stokes et al., 2020). *In silico* models could further support individualized dosing strategies by predicting PK/PD behavior, toxicity risk, and the likelihood of resistance emergence. This precision medicine framework has the potential to reduce unnecessary broad-spectrum antibiotic use, strengthen antimicrobial stewardship, and improve clinical outcomes.

## 12 Conclusion

In summary, *in silico* drug discovery pipelines are fundamentally transforming the landscape of antibiotic research by providing data-driven, scalable, and evolution-aware strategies for combating antimicrobial resistance. The integration of genomics, AI, and systems biology offers a powerful framework for accelerating target identification, optimizing lead compounds, improving translational success, and extending the therapeutic lifespan of antibiotics. Successful examples such as AI-assisted antibiotic discovery and structure-guided inhibitor development demonstrate that computational approaches are no longer merely supportive tools but are becoming central components of modern antimicrobial innovation. However, the successful implementation of these digital-first discovery models depends on several critical factors, including the generation of high-quality and unbiased datasets, improved model transparency, rigorous experimental validation, and strong ethical and regulatory governance. Challenges related to data bias, limited generalizability, explainability of AI models, and clinical translation must be addressed to ensure reliable and clinically relevant outcomes. As these computational platforms continue to evolve, they are expected to shift antibiotic development from a reactive and empirically driven process toward a proactive, precision-guided discipline capable of anticipating resistance and designing durable therapeutic solutions. This transformation represents one of the most promising strategies for addressing the global AMR crisis and safeguarding the future effectiveness of antimicrobial therapy in the twenty-first century.
